# Supplementary figures and images for: Zebrafish Ski7 tunes RNA levels during the oocyte-to-embryo transition
Source: PLoS Genet. 2021 Feb 18;17(2):e1009390. doi: 10.1371/journal.pgen.1009390 (PMC7924785; doi:10.1371/journal.pgen.1009390)

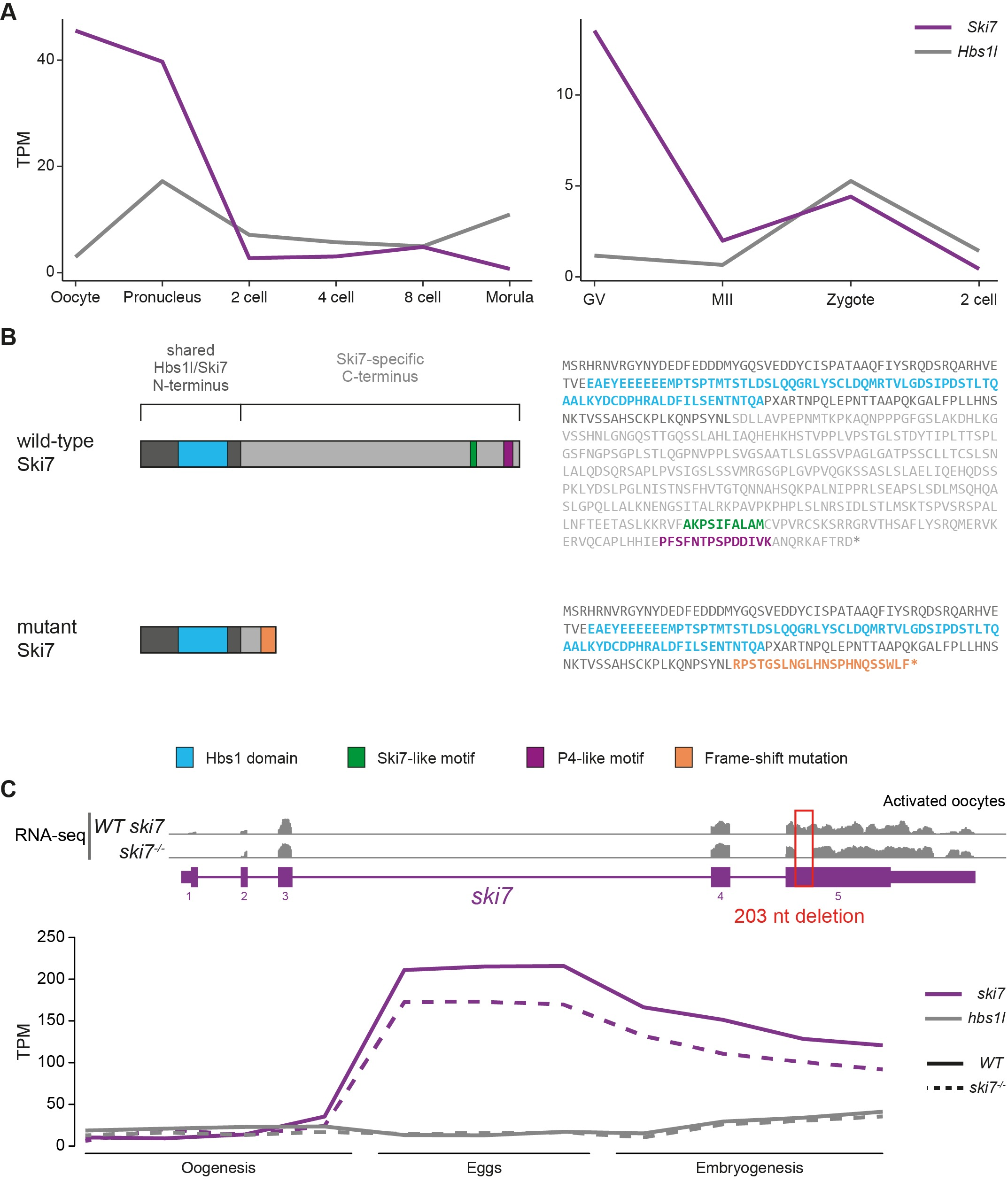

Supplement: S1 Fig — (A) Expression levels (TPM) of mouse Ski7 (purple) and Hbs1l (gray) during early embryogenesis. Left: Hendrickson PG, et al 2017; right: Yu C, et al, 2016. (B) Zebrafish Ski7 mutant protein lacks the conserved Ski7-like and P4-like motifs. Schematic (left) and amino acid sequence (right) of wild-type and mutant Ski7 proteins. Specific domains and motifs are highlighted. The mutant protein contains the shared N-terminus from Hbs1l but lacks the Ski7-like and P4-like motifs due to a premature stop codon. (C) Hbs1l mRNA levels remain unchanged in ski7-/- mutants. Screenshot of RNA-seq reads over the ski7 gene locus of mutant and wild-type samples. Red box indicates the 203-nt deletion in the mutants (top). TPM levels of ski7 (purple) and hbs1l (gray) in WT (straight line) and ski7-/- (dotted line) across the entire time-course show no differences in expression for hbs1l. (TIF) [file pgen.1009390.s001.tif]

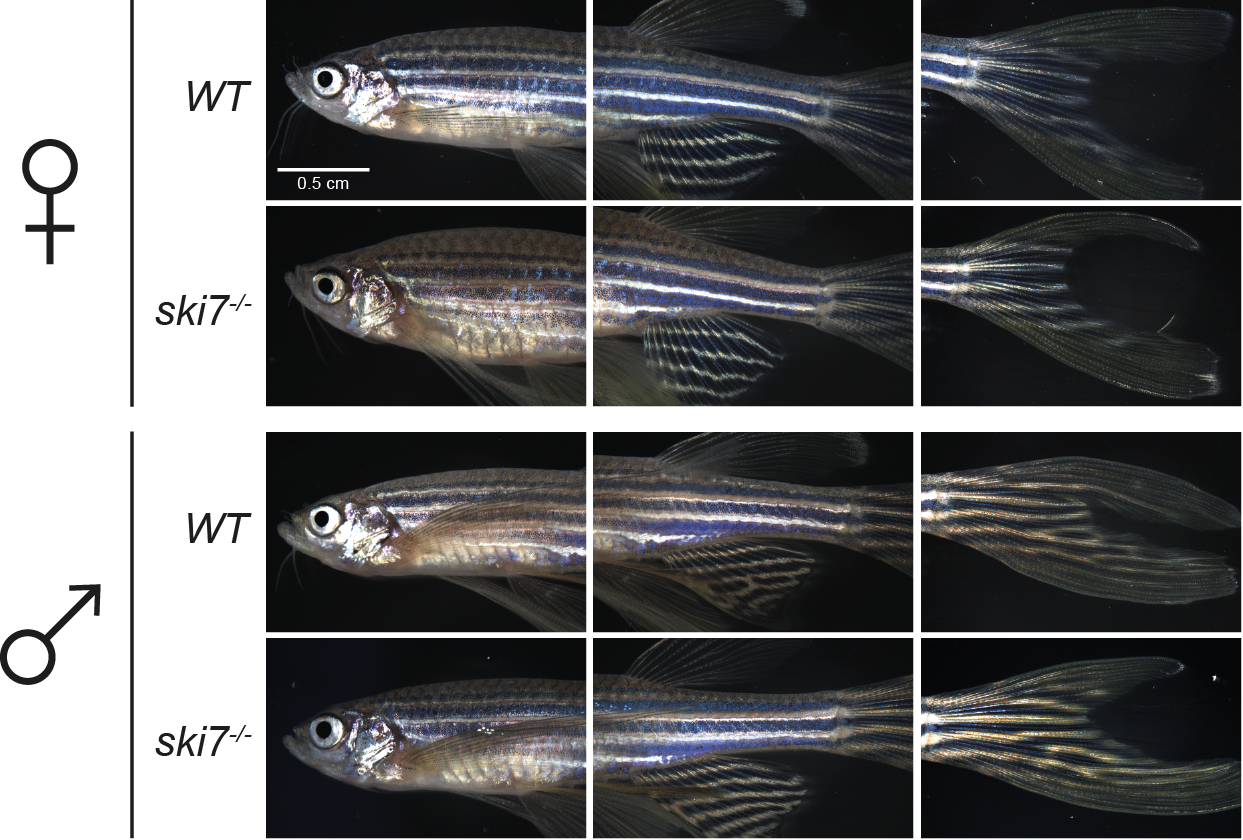

Supplement: S2 Fig — Representative images of wild-type and ski7-/- mutant female (top) and male (bottom) fish. No morphological differences are observed. (TIF) [file pgen.1009390.s002.tif]

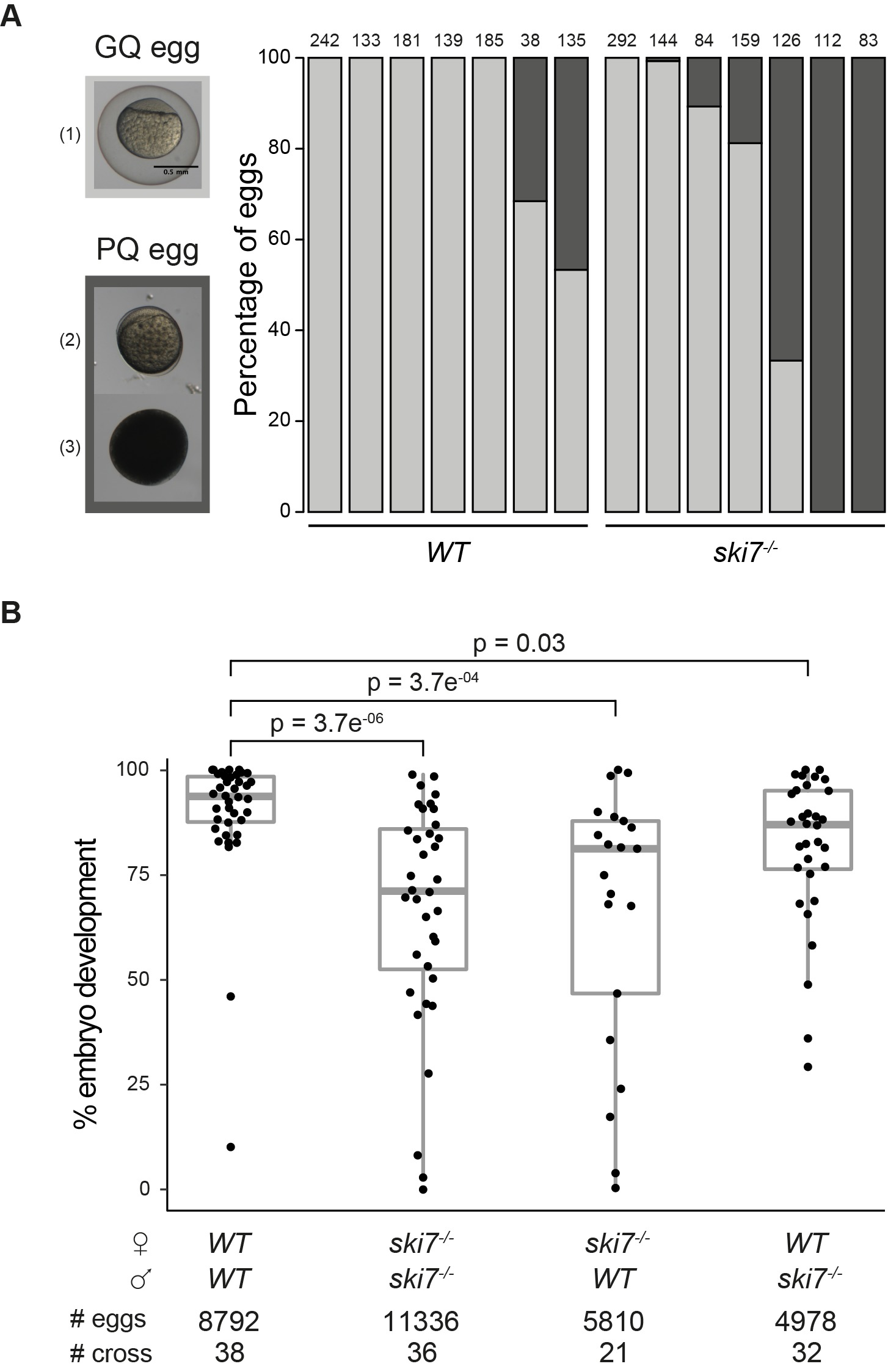

Supplement: S3 Fig — (A) Representative images and quantification of eggs of good quality (GQ) and poor quality (PQ) at 30 minutes after egg-laying. (1) Good egg quality, (2) egg without chorion elevation, and (3) opaque egg. Every bar represents an individual female. Numbers on top of the bars indicate the number of eggs. (B) Percentage of developing embryos from wild-type, mutant, and reciprocal crosses. P-adjusted from Kruskal-Wallis with Dunn’s comparison test (comparisons were performed against the cross of WT male with WT female). WT-WT and ski7-/—ski7-/- crosses are from main Fig 1D. (TIF) [file pgen.1009390.s003.tif]

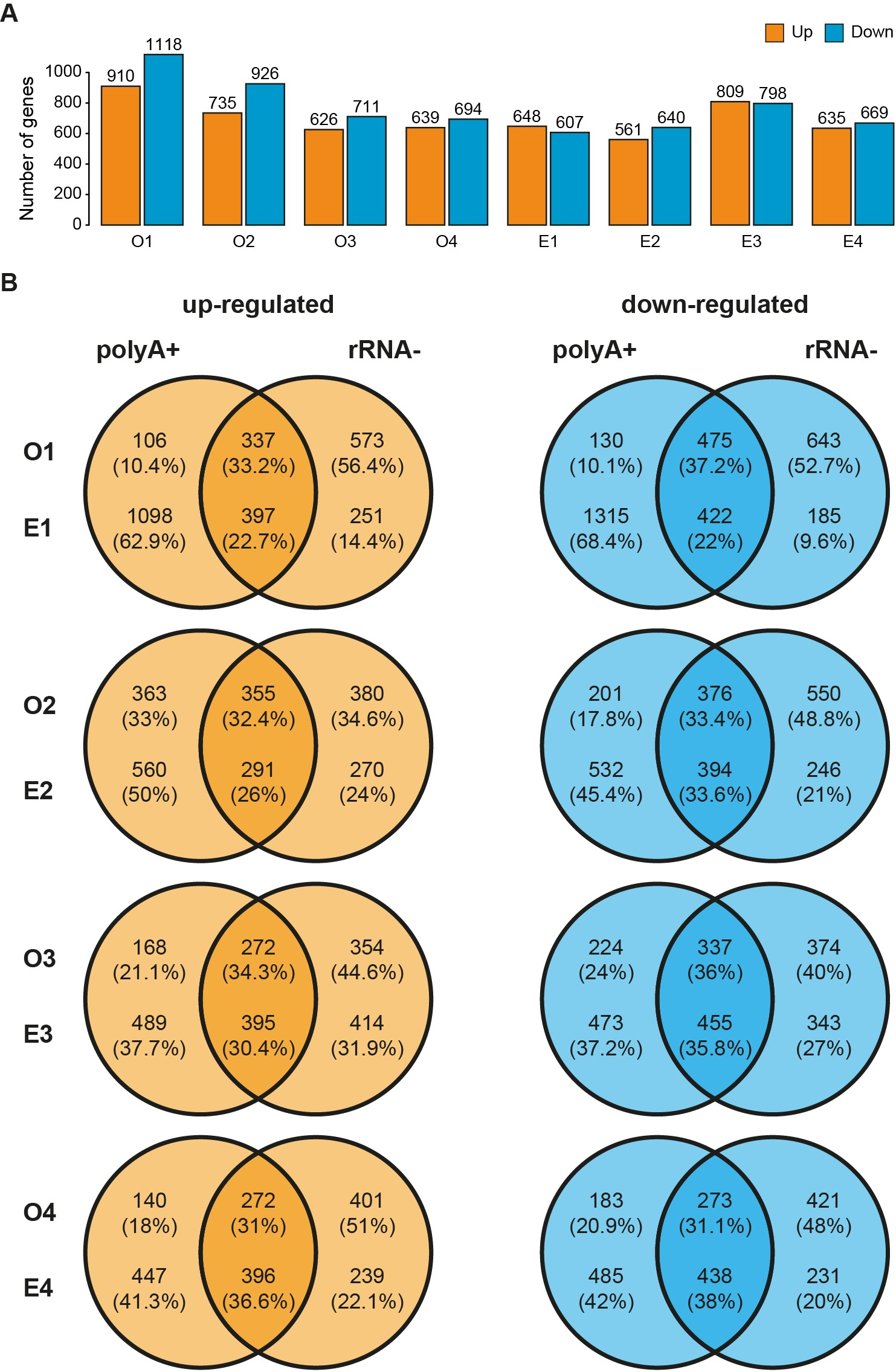

Supplement: S4 Fig — (A) Number of up- and down-regulated genes in ski7-/- mutants during oogenesis (O1 –O4), and embryogenesis (E1 –E4) based on rRNA-depleted mRNA enrichment. (B) Venn diagrams of the number and percentage (in brackets) of DEGs obtained by polyA+, rRNA-, or both mRNA enrichment methods. (TIF) [file pgen.1009390.s004.tif]

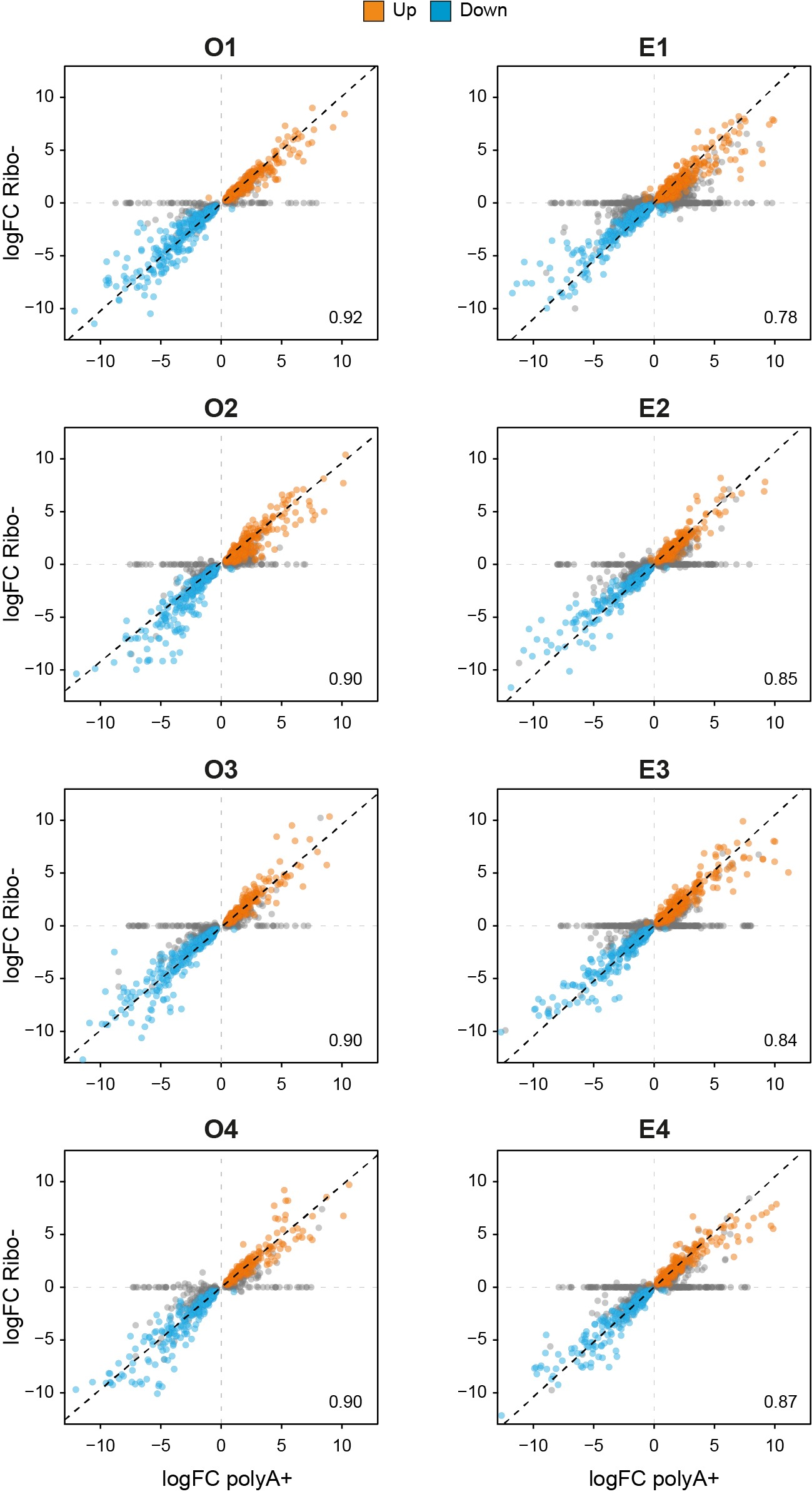

Supplement: S5 Fig — Scatter plots of polyA+ and rRNA- log fold changes of DEGs identified by polyA+ RNA during oogenesis and embryogenesis. Colored dots represent individual genes that were classified as significantly differentially expressed in both methods. The number (bottom right corner) indicates Spearman’s ran correlation coefficient. (TIF) [file pgen.1009390.s005.tif]

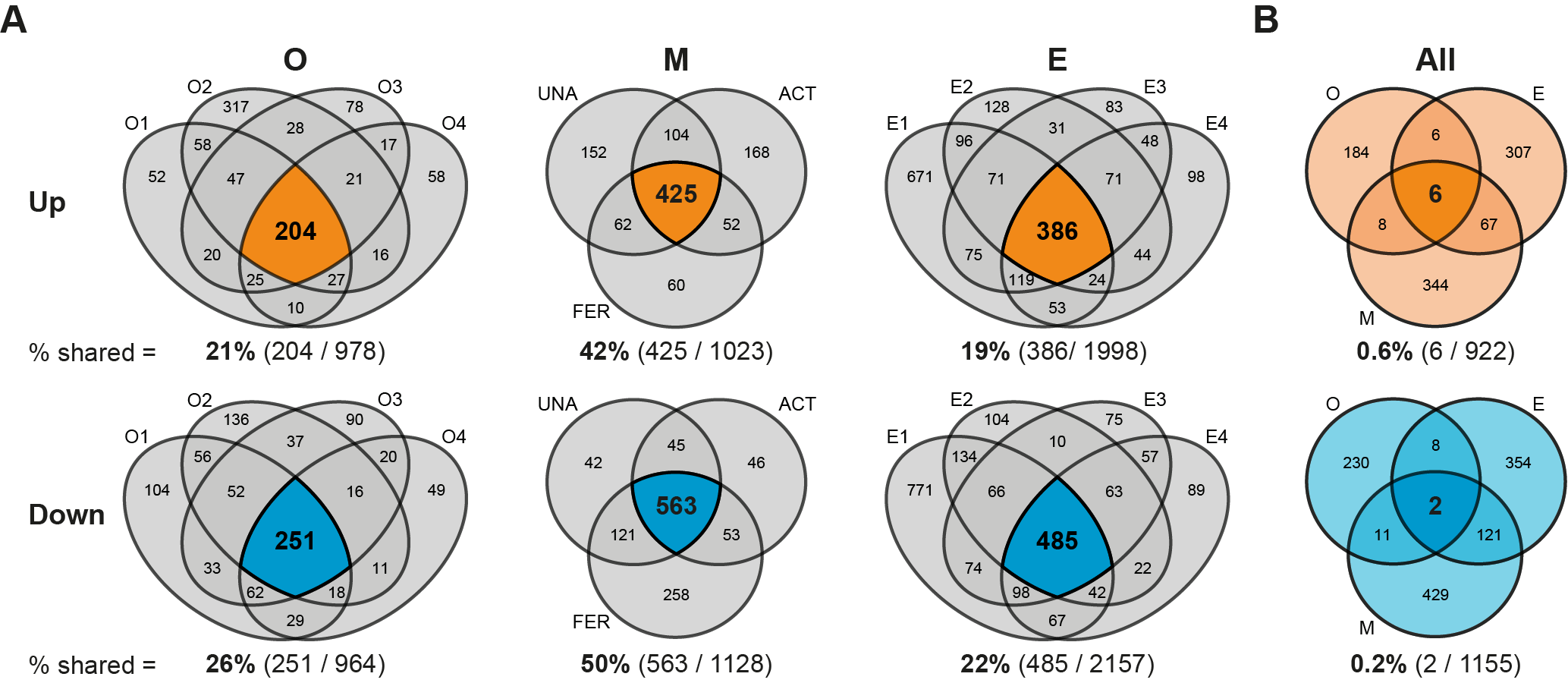

Supplement: S6 Fig — (A) Number of up- and down-regulated genes in ski7-/- mutants during oogenesis (O), in mature eggs (M), and embryogenesis (E). (B) Venn diagrams of differentially expressed genes for the three periods. Note that there is almost no overlap of genes between all three periods. (TIF) [file pgen.1009390.s006.tif]

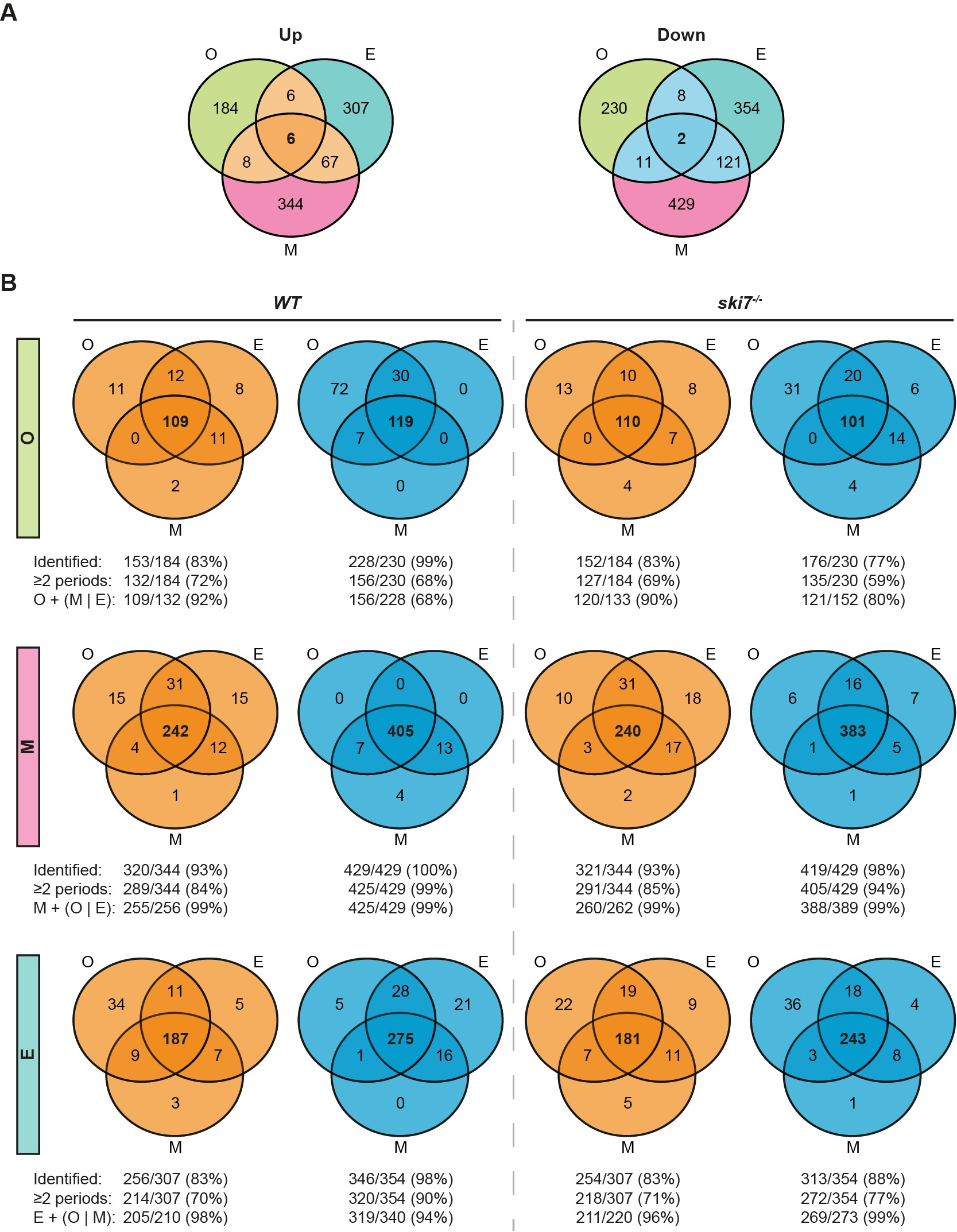

Supplement: S7 Fig — (A) Venn diagrams of the overlap of DEGs from the three periods from S6B Fig. (B) Venn diagrams of the overlap of ‘period-specific’ DEGs (differentially regulated in all stages of a given period) during the oocyte-to-embryo transition in WT and ski7-/- samples. The numbers below refer to the expression in wild type (left) or ski7-/- (right). Identified = expressed in wild type or ski7-/- in at least one period; ≥2 periods = expressed in wild type or ski7-/- in at least two periods; X + Y|Z = expressed in the period of origin (X) and at least one other period (Y and/or Z). (TIF) [file pgen.1009390.s007.tif]

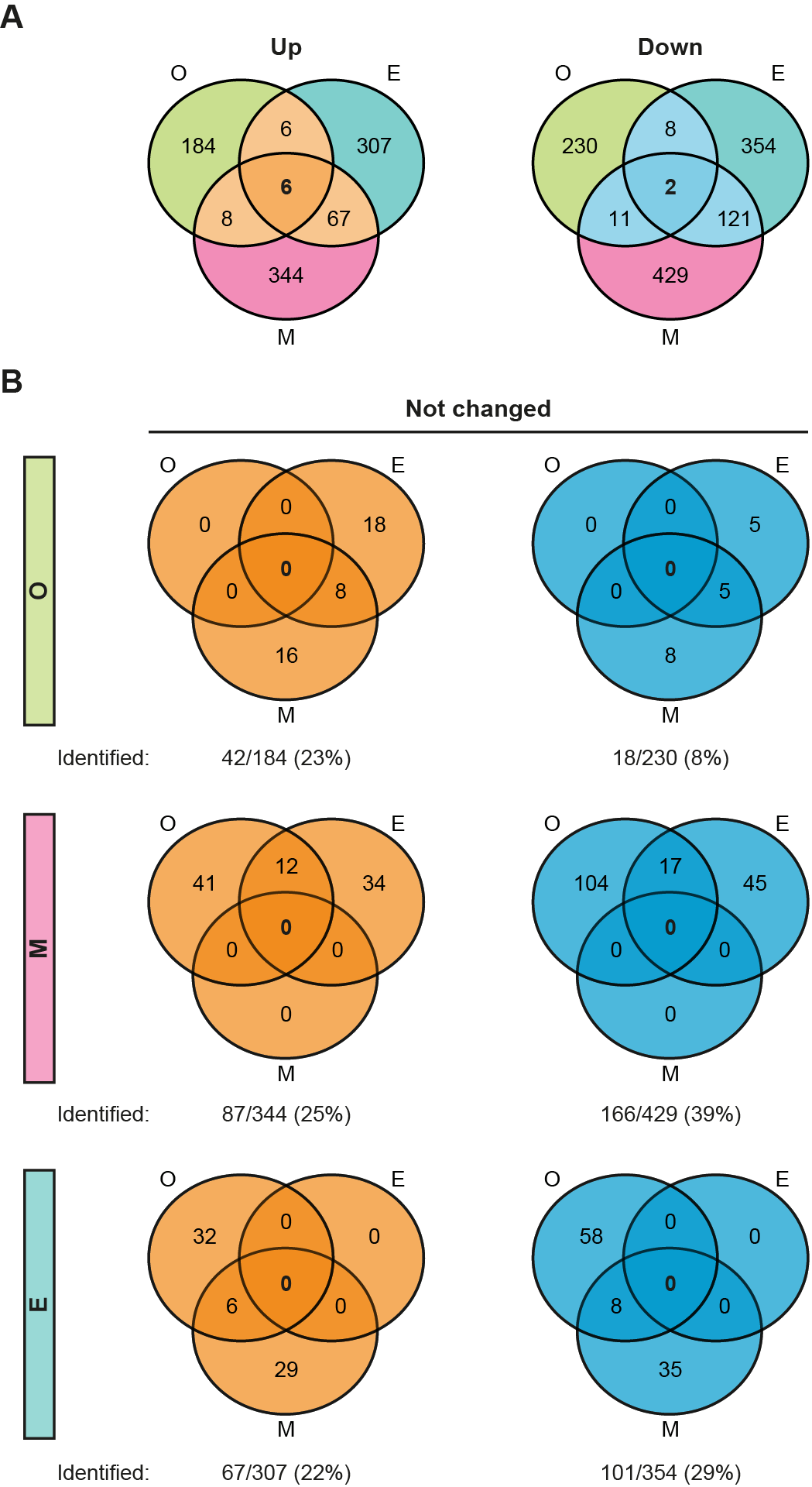

Supplement: S8 Fig — (A) Venn diagrams of the overlap of DEGs from the three periods from S6B Fig. (B) Venn diagrams of the overlap of ‘period-specific’ DEGs (differentially regulated in all stages of a given period) compared to unchanged genes in the other periods. The numbers below refer to DEGs of the indicated period identified as unchanged in other periods. (TIF) [file pgen.1009390.s008.tif]

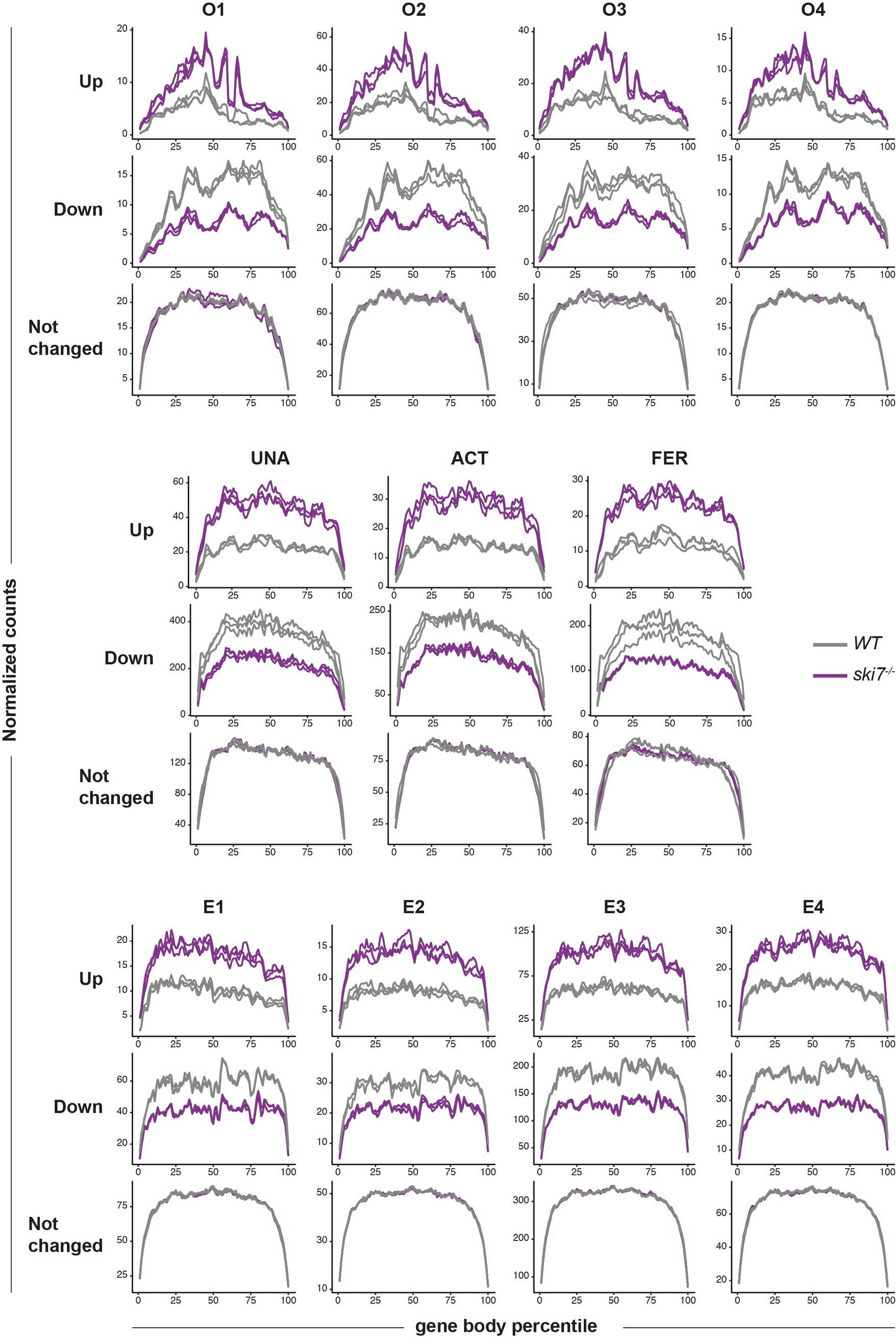

Supplement: S9 Fig — Metagene profiles of up-regulated, down-regulated, and unchanged genes of wild-type (gray) and mutants (purple) at all stages during the oocyte-to-embryo transition. (TIF) [file pgen.1009390.s009.tif]

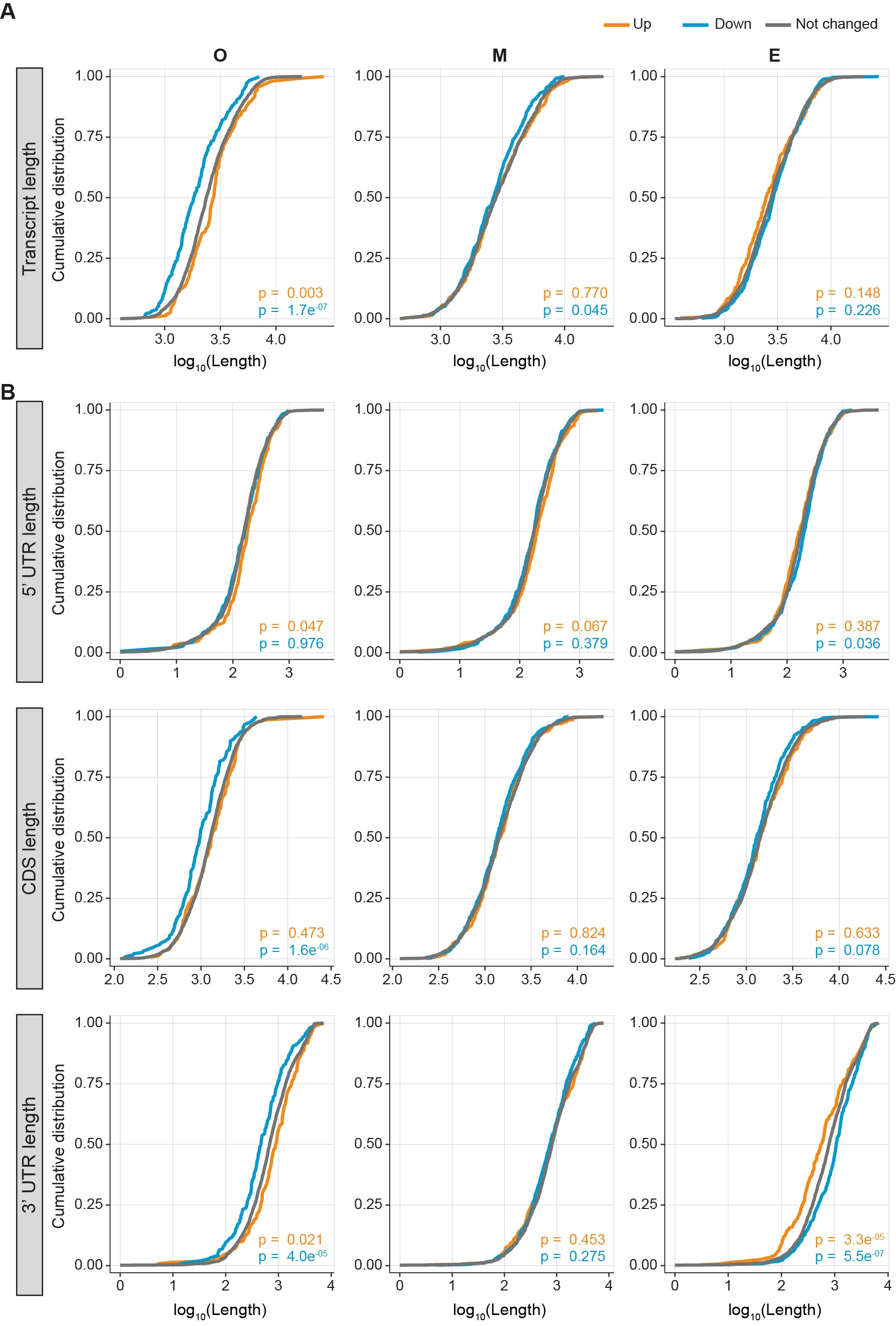

Supplement: S10 Fig — Cumulative distribution of transcript lengths of up-regulated (orange), down-regulated (blue) and unchanged (gray) genes per period. (A) Analyses of the length of full transcripts shows that down-regulated genes during oogenesis tend to be shorter while up-regulated are longer (Kolmogorov-Smirnov p = 1.7e-07 & 0.003, respectively). (B) Analyses of the length per transcript region (5’ UTRs, CDSes and 3’ UTRs). Note that the biggest difference in down-regulated genes during oogenesis is observed in CDSes and 3’ UTRs (Kolmogorov-Smirnov p = 1.6e-06 & 4.0e-05, respectively), and 3’ UTRs in up-regulated genes (Kolmogorov-Smirnov p = 0.021). (TIF) [file pgen.1009390.s010.tif]

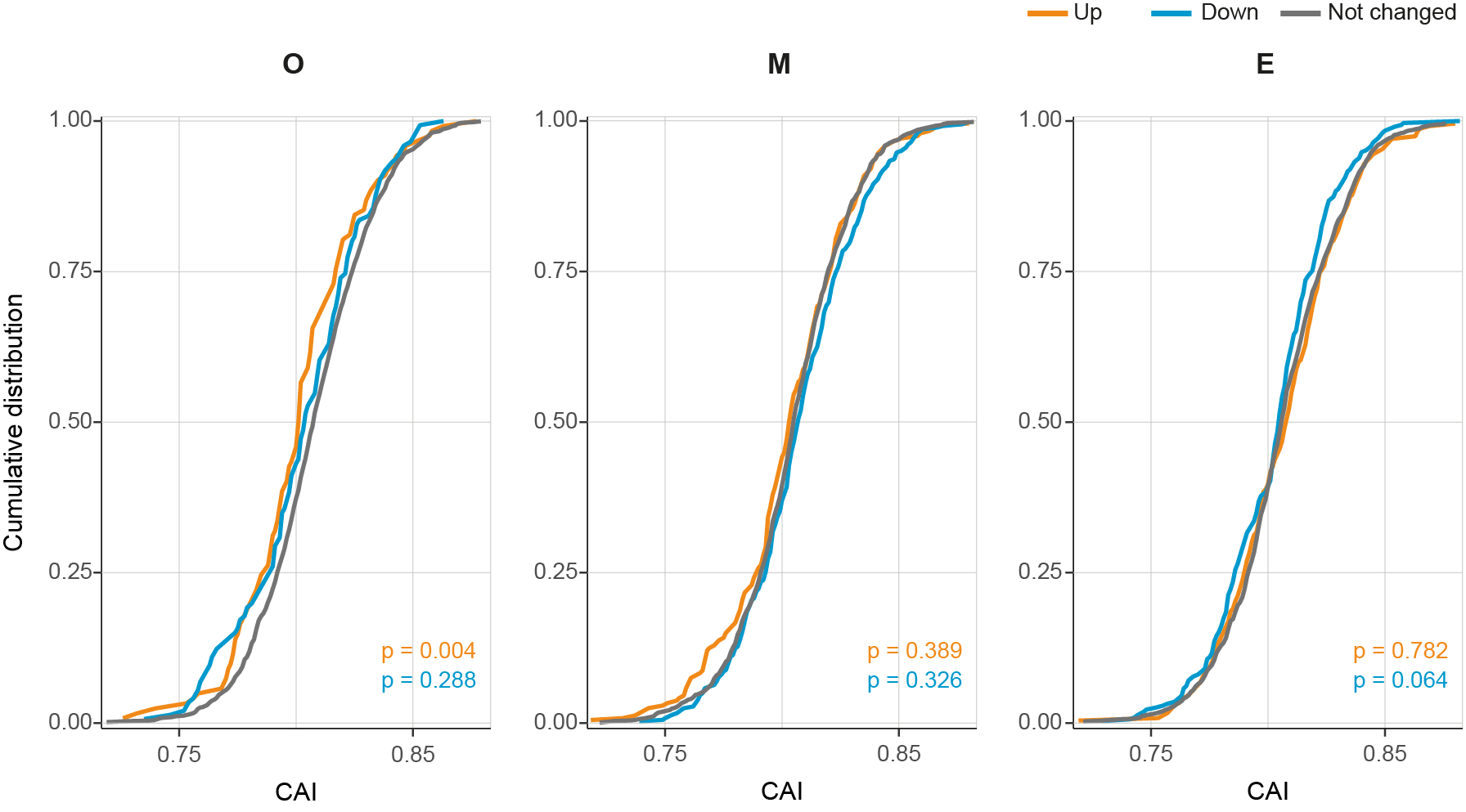

Supplement: S11 Fig — Cumulative fraction of the usage of synonymous codons in DEGs (up-regulated: orange, down-regulated: blue) and unchanged genes (gray) per period as measured by codon adaptation index (CAI). P-values from Kolmogorov-Smirnov test. (TIF) [file pgen.1009390.s011.tif]

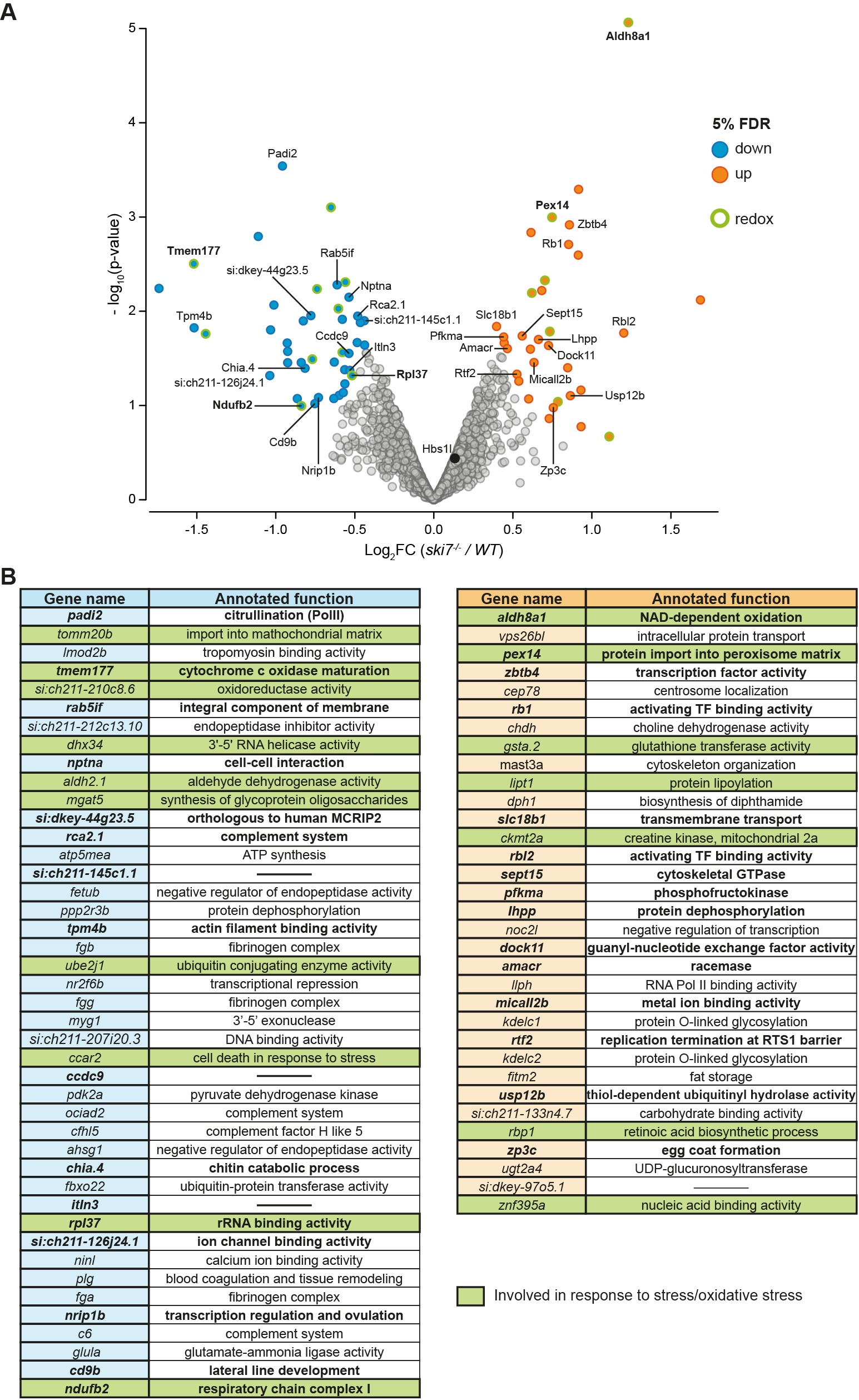

Supplement: S12 Fig — (A) Volcano plot of proteins identified by tandem mass tag mass spectrometry (TMT-MS) from wild-type and ski7-/- mutant embryos at 4 hours post-fertilization. Significantly up- and down-regulated proteins are coloured in orange and blue, respectively. Factors involved in response to stress/oxidative stress are highlighted in green. Labelled dots highlight proteins for which the mRNA was also identified as differentially regulated by RNA-seq. In bold, oxidative stress proteins that were also identified by RNA-seq. (B) List of all significantly up- and down-regulate proteins (indicated by gene name) from the TMT-MS analyses. Differentially expressed proteins for which the mRNA was also identified as differentially expressed by RNA-seq are highlighted in bold. Genes that have been associated with stress response or oxidative/reductive stress are highlighted in green. (TIF) [file pgen.1009390.s012.tif]

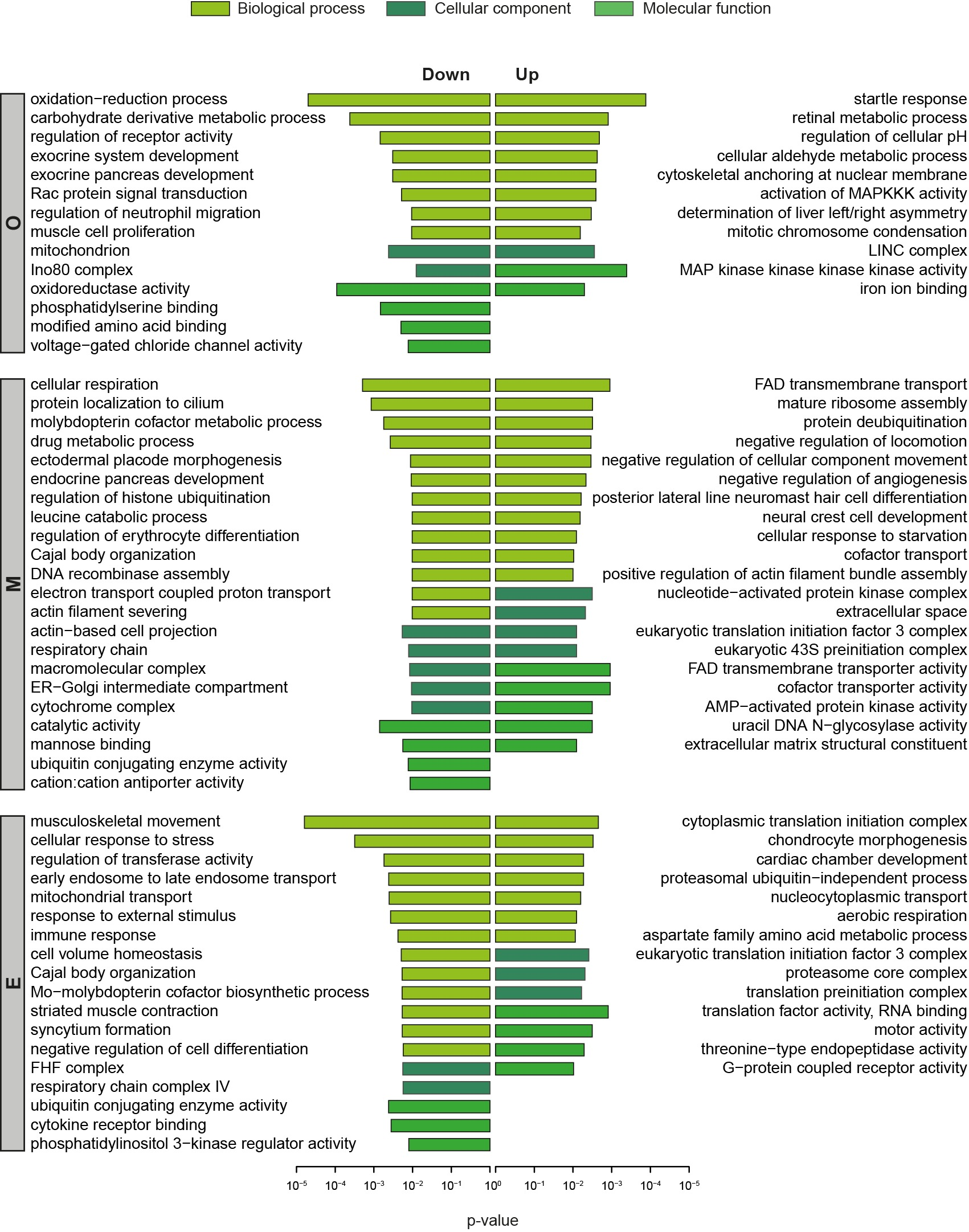

Supplement: S13 Fig — GO terms enriched in the overlapping set of DEGs from each period (O = oogenesis, M = mature eggs, E = embryogenesis). (TIF) [file pgen.1009390.s013.tif]

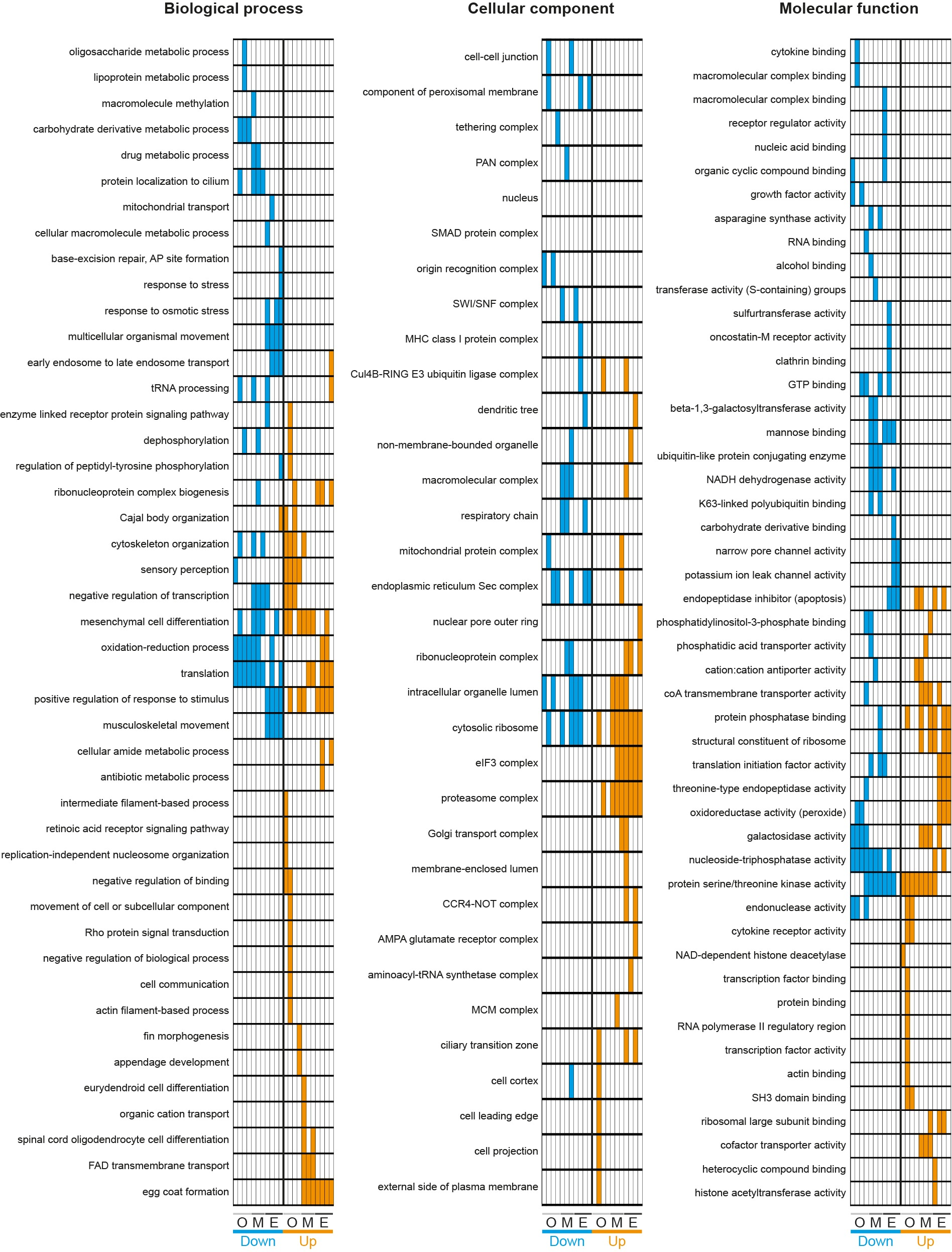

Supplement: S14 Fig — GO terms enriched in DEGs at each stage of the time-course experiment during the oocyte-to-embryo transition. O = oogenesis, M = mature eggs, E = embryogenesis. (TIF) [file pgen.1009390.s014.tif]
