# Supplementary material for: Zebrafish Ski7 tunes RNA levels during the oocyte-to-embryo transition
Source: PLoS Genet. 2021 Feb 18;17(2):e1009390. doi: 10.1371/journal.pgen.1009390 (PMC7924785; doi:10.1371/journal.pgen.1009390)
Supplement: Attachment — Submitted filename: 104890_1_rebuttal_1838353_qf97fl.pdf [file pgen.1009390.s031.pdf]

Thank you very much for providing reviews via Reviews Commons for our manuscript entitled "Zebrafish Ski7 tunes RNA levels during the oocyte-to-embryo transition". We were very pleased with the fast return of reviewers' comments and also with the high scientific quality and depth of the reviews. We would like to thank both reviewers for their constructive and very valuable feedback for our manuscript.

While both reviewers highlighted the importance of our work since the in vivo function of Ski7 in animals had not been analysed before (reviewer 1: '*The authors use Zebrafish as a model, but this is the first report of Ski7 function in any animal. Thus, the results are widely interesting*.'), they also raised concerns regarding the conclusions we draw from the data we present. In the past 3 months, we have obtained additional data (both in terms of RNA-seq data as well as a new transgenic Ski7 rescue line) that have allowed us to address these concerns and further strengthened our manuscript.

In the following we address the comments of the reviewers in a point-by-point manner.

Reviewer #1 (Evidence, reproducibility and clarity (Required)):

The authors describe the functional analysis of Ski7 in zebrafish, a cofactor of the exosome that is thought to be important for 3' to 5' mRNA decay. The authors use Zebrafish as a model, but this is the first report of Ski7 function in any animal. Thus, the results are widely interesting. The authors noticed that Ski7 expression peaks in early development in both zebrafish and mice, and thus focus on this stage. They perform transcriptome sequencing at 11 different stages of development, from oogenesis to E4 stage embryos. The key conclusion is that Ski7 targets hundreds of mRNAs for degradation, but that different mRNAs are targeted at different stages. The authors also conclude this function is conserved between yeast and animals. Although the work is potentially interesting, the authors' conclusions are not fully supported by their data and their statistical analyses. Thus, the text of the manuscript should be adjusted before publication.

Thank you very much for your very helpful feedback on our manuscript. The specific points you raised below, and specifically your valid concern regarding our conclusions drawn from the RNA-seq analyses, have helped us to improve our manuscript. As outlined below, we have added additional analyses and revised our manuscript accordingly to address these concerns.

****Major comments:****

1.Because of a number of technical issues with the RNAseq analysis the authors conclusion that different low-expression genes are targeted at different stages is not convincing. A substantial increase in the number of samples (and perhaps library size) would be likely needed to definitively show this.

There have been several studies published on how many replicates and how many reads one needs. See for example https://pubmed.ncbi.nlm.nih.gov/24319002/ The consensus is that the more replicates, the better. For homogeneous cell populations, three replicates is the bare minimum, but comes at the cost of significant false negatives. More heterogeneous samples require more replicates. Here, RNAseq was done in triplicate (for most time points). This is sufficient to detect some affected genes, but does not provide sufficient statistical power to identify all affected genes and avoid false negatives.

Thank you very much for raising this concern. We fully agree with you that an increase in the number of independent replicates would be ideal, yet given that we are dealing with 2 genotypes and 11 stages, collecting this data set in independent biological triplicates has already resulted in 66 independent samples. Given the overall good agreement between independent replicates for individual time points (see **Sup. Data 3**) and triplicates being generally accepted for differential gene expression analyses, we decided to use 3 biological replicates for the RNA-seq experiments as it has

been the standard in many publications. Thank you for pointing out that two specific samples had only been included as duplicates. We re-sequenced these two libraries that previously failed to contain sufficient reads, such that now all time points in both genotypes are represented by three replicates. In addition, we have switched from using DESeq2 to EdgeR with more stringent criteria for calling differentially expressed genes (now using a threshold of at least 10 CPM per library in at least three replicates per period), and increased sequencing depth for each sample to ~10 Million reads in light of your suggestion (see below) to obtain a more robust set of differentially expressed genes. We have reanalyzed all of our data with the larger RNA-Seq data set and revised our manuscript accordingly. Overall, our re-analyses allow us to make the following revised conclusions: 1) there are similar numbers of up- and down-regulated genes in ski7 mutants; 2) DEGs within periods (during oogenesis, in mature eggs, during early embryogenesis) show a significantly larger overlap than expected by chance when compared to unchanged genes of similar expression levels (**Fig. 3E**); 3) DEGs between periods remain largely different (**Fig. 3E**, **Sup. Fig. 6-8**), supporting our initial conclusion that there is a time-specific component in Ski7-mediated regulation that is not due to the differentially expressed genes being only expressed during those stages. Yet in light of the clear overlap observed between DEGs within periods we have updated our text and also title of this paragraph. We have updated our manuscript both in terms of Figures and text accordingly.

The authors state that "Libraries that had fewer than 0.5 million reads were not considered for the analyses". The size of the analyzed libraries is not stated. Unless libraries were very much larger that 500,000 this may further limit the statistical power. The consensus of published studies is that 10 mllion reads per library is more appropriate. The authors should include a table with the size of each library.
Thank you for your suggestion. We have now increased the sequencing depth of all libraries to have on average at least 10M reads. We have also added a table with the number of sequenced reads per sample (**Sup. Data 3**). In addition, we also performed a saturation test in which we down-sampled the same number of aligned reads per million (from 1 to 10M) for each replicate and used that to perform differential expression analyses. The number of reads was plotted against the number of identified DEGs. As seen below in the graphs, for the majority of samples, increasing library sizes >7-8M caused only a slight increase in the number of newly identified DEGs, indicating that deeper sequencing would not identify many more DEGs.

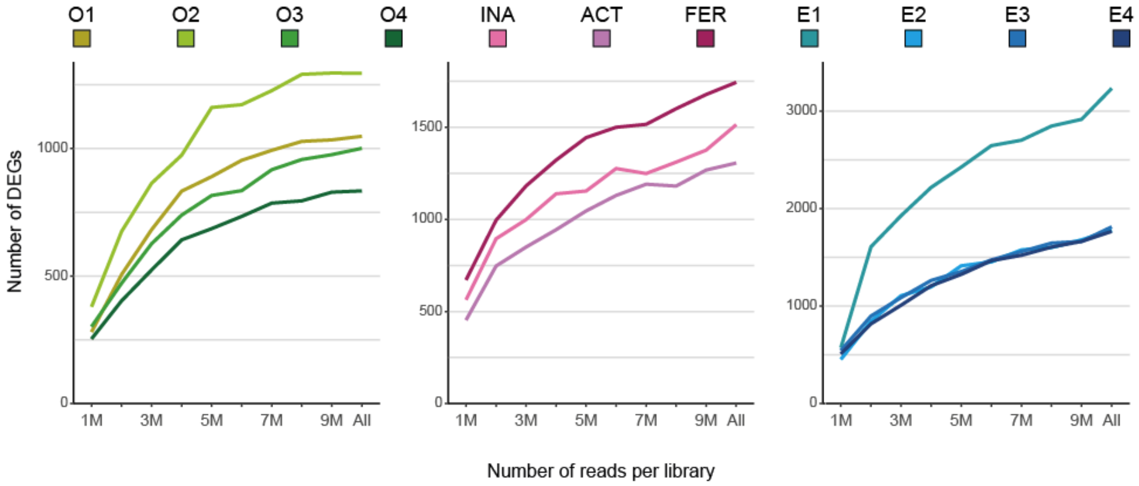

Remarkably, the authors find that mostly rare mRNAs are affected with a median of about 2 transcripts per million (Figure 4A). The ability to reliable detect and quantitate mRNAs is even more problematic for low expression genes.

Because of these limitations the DEG analysis is likely to contain a significant number of false negatives, i.e. there are many mRNAs that are affected in reality, but the RNAseq does not provide statistically significant evidence to identify them.
The presence of false negatives itself is not a problem, but it becomes a problem when the authors ignore their existence and make the incorrect assumption that if DEG does not provide evidence for a gene being affected, then that is proof that gene is not affected. However, absence of evidence for a difference can not be treated as evidence for absence of a difference, which is exactly what the authors do in figure 3E. The addition of an (arbitrary) 2-fold cutoff worsens the problem further. The genes in the gray areas of figure 3E include any gene that is a false negative in one of the stages, plus any genes that are affected 1.99 fold at one stage but 2.01 fold at another. I have more confidence in the PCA analysis of Figure 3A. Panels 2, 3, and 4 of the PCA each show that ski7 has an overall similar effect on the transcriptome. For example, for the oogenesis samples PC2 cleanly separates all of the wt samples from all of the mutants samples and the effect of ski7 is in the same direction and magnitude for each stage of oogenesis. Thus, PCA indicates that the transcriptome is altered in the same way at each stage, which is opposite of the authors conclusion "Ski7 regulates transcripts in a time-specific manner" (in bold heading of the results).
Thank you very much for your comment. We agree with the reviewer that including a fold change cutoff is not necessarily the best strategy to identify all DEGs. In our initial analysis, we identified all DEGs as being confidentially different based on their fold change. However, as the reviewer points out, comparison among time points would not be correct under these circumstances. We thus decided to now consider all DEGs (FDR≤0.05) regardless of their fold change. In addition, we performed the comparison of DEGs against expressed genes with the same number of genes and matched-expression levels to the DEG sets. This new analysis revealed that within the three periods (oogenesis, eggs, and embryogenesis) DEGs are indeed shared at a higher frequency than what is expected by chance (**Fig. 3E**). However, DEGs between different periods still persisted to be distinct (almost no overlap) (**Fig. 3E, Sup. Fig. 6-8**). In light of the results of this revised analysis, we have changed the title of this section to "Ski7 regulates transcript levels during the oocyte-to-embryo transition".

2.The authors use poly(A)+ mRNA in their transcriptome sequencing and therefore any in- or decreases detected reflect the poly(A)+ fraction only. Remarkably, yeast Ski7 is thought to target mRNAs for further degradation after the poly(A) tail is removed. One possibility is that the authors conclusion that "zebrafish Ski7 acts similarly to yeast Ski7 in contributing to 3'- to-5' mRNA degradation" is incorrect. If zebrafish ski7 targets polyadenylated mRNA, that would be a fundamental difference with the yeast function. Another possibility is that the transcriptomic effects the authors report are dominated by indirect effects. The authors suggest that they detect mainly direct effects because more genes are up in the mutant than down, but the bias towards upregulation is weak. The ratio of upregulated genes relative to downregulated genes varies between 1.2 and 1.6. The authors need to include a discussion on the use of poly(A)+ RNA in their interpretation of the data.
Thank you for raising this point. Exactly for the reason that you brought up (yeast Ski7 is thought to target mRNAs after the poly(A) tail has been removed), we had in parallel also generated rRNA depleted (rRNA-) libraries for oogenesis and embryogenesis periods. We had not included them in our initial submission for two reasons: 1) we do not have the corresponding rRNA- samples for mature eggs, and 2) we had observed a general agreement between the polyA and rRNA- datasets, thus felt that adding the not fully complete dataset would not add much. However, we agree with your comments and thus decided to add the rRNA- data to the revised manuscript. We analyzed it in the same manner as our poly(A)+ libraries, performed the comparison between poly(A)+ and rRNA- (correlation plots (**Sup. Fig. 5**) and identified the number of shared DEGs per time point (**Sup. Fig. 4**). These analyses reveal that the two datasets largely agree and follow a similar trend. The main reasons why we focus in the main part of our analysis on the poly(A)+ libraries are that 1) we have also the intermediate stages (mature eggs) during the oocyte to embryo transition, and 2) mRNAs were sequenced deeper in the polyA+ libraries.

3.Figure 5B and C are missing untreated controls that are needed to substantiate the claim that absence of ski7 confers increased resistance to DTT.
Thank you for your comment. We already had the data for the untreated controls and have added the pictures and quantification to the figure (**Fig. 5B-C**).

4.The description of ski7 mutant phenotypes is incomplete. The abstract states that ski7 mutant fish "developed into morphologically normal adults" and one of the headings in the results is "Zebrafish Ski7 is not essential for survival". The methods section does not describe how morphology or survival were assessed and no results are shown. It is thus not clear how careful the authors looked for subtle phenotypes. It would be interesting to add a discussion on trichohepatoentic syndrome. This human disease is caused by mutation in hSKI2 (SKIV2L) or hSki3 (TTC37), but patients with mutations in hSKI7 have not been described.
We appreciate your concerns regarding the viability and adult phenotype of the *ski7*$^{-/-}$ fish. We did not perform an in-depth phenotypic characterization of adults, yet have not noticed any morphological phenotypes or a decrease in survival for the past 3 years since we have these homozygous mutant fish in our facility. Fish in our facility are generally kept up to 2 years of age, and we have not observed any decrease in viability of ski7 mutants during this time-frame. We have now included a representative picture of a female and male adult fish as a supplementary figure to indicate that there are no apparent morphological differences (**Sup. Fig. 2**).
Your point regarding the other components of the Ski complex is very interesting, yet we feel beyond the scope of our manuscript where we focus on the analysis of Ski7 due to its high expression during the egg-to-embryo transition. The other complex members do not show a peak in expression in the mature egg in zebrafish. Moreover, based on our pull-down experiment and the published data based on human cells (Kalisiak, *et al*. 2016), vertebrate Ski7 does not seem to strongly interact with the Ski complex. We therefore feel that analysis of the other Ski complex members warrants a separate analysis and is beyond the scope of our manuscript.

5.The * in figure 5C indicates that there is a (barely) significant effect of ski7 at 2hpf. The text "ski7-/- embryos were more resistant to reductive stress than wild-type embryos (Fig. 5B, C). This effect was most pronounced between two to four hours post-fertilization though persistent throughout the time course" does not reflect that. Either there are missing asterisks in the figure or a more accurate text would be "This effect was barely significant at 2h, and no significant effect was detected at later time points".
Thank you for pointing out this inconsistency regarding text and data. With the addition of the untreated controls, we performed the statistical test (Kruskal-Wallis with Dunn's multiple comparison) per time point. This analysis revealed a consistent difference in wt embryos with/without DTT (yet as observed before, no such difference in ski7 mutants). We have modified the text and Figure (**Fig. 5B-C**) accordingly.

Minor comments

6.The title of figure 4 "Genes degraded in a Ski7-dependent manner are lowly expressed and degraded from 3'-to-5'" should be shortened to ""Genes degraded in a Ski7-dependent manner are lowly expressed" since the data supporting the 3' to 5' degradation are not in this figure but instead in Figure S3. Even better would be "genes overexpressed in the ski7 mutant are lowly expressed" in recognition of the fact that some might be indirect effects.
Thank you for your comment. We now modified figure 4 and included the supplementary data as a panel in the main figure **(Fig. 4C**). We agree that the new 4C panel is better suited to display our conclusions regarding the 3'-5' degradation.

7.The authors use "co-immunoprecipitation" to describe their pull down experiments, but since there are no antibodies involved the more generic term "pull down" is more appropriate.
Thank you for highlighting this. We modified the text to indicate pull-down where appropriate.

8.The referencing seems limited and biased toward recent papers. Often one recent reference is given when multiple papers have show this. Especially striking on page 12 the sentence "In mammals, it is well established...." has one reference from 2020. A single reference from this year suggests it is not (yet) well-established.
Thank you for bringing up this point. In the specific example you mentioned we have changed the wording to "it has been shown" to better reflect the status of the field. Ski7 has only recently been identified outside yeast, thus most references mentioned here are indeed biased to be rather recent. We have now also included a new reference to a bioRxiv paper that just came out while our paper has been under review (Blatt et al., 2020). We also went carefully through the manuscript and added other appropriate references that support our statements.

Reviewer #1 (Significance (Required)):

Significance

see above

REFEREES CROSS COMMENTING

I agree with the comments from the other reviewer . His major comment that the authors have not shown that the observed effects are indeed caused by the ski7 mutation is important, but had escaped me.
We have addressed this important concern by generating a rescue line for our *ski7* mutant (transgenic expression of Ski7-GFP). We found that this line fully rescues the fertility defect of our *ski7* mutant, which allows us to draw the important conclusion that the compromised fertility and thus decreased fitness of the line is indeed due to lack of Ski7 and not an unrelated potential off-target/background effect of the *ski7* mutant line. We have included this new data in Figure 1 (**Fig. 1D**)**,** and updated the text and methods accordingly.

Reviewer #2 (Evidence, reproducibility and clarity (Required)):

****Summary:****

The authors studied the zebrafish homolog of yeast Ski7, an adaptor protein that functions with the Ski complex to activate the cytoplasmic exosome to perform 3'-5' RNA decay activities. They generated a global Ski7-null zebrafish line using CRISPR methods and tested the impact on fertilization, development, and RNA levels. Their key conclusions are as follows:

• Ski7 is more highly expressed in eggs and early embryos than at other developmental stages.

• Ski7-null fish, particularly females, are subfertile as indicated by a lower percentage fertilization of spawned eggs, but once fertilized, embryo development is completely normal.

• Based on IP-mass spec studies, Ski7 protein interacts with cytoplasmic exosome components as in yeast and human cells.

• Loss of Ski7 causes changes in gene expression in oocytes, eggs, and embryos, but more genes are differentially expressed in egg stages and these are more often up- than downregulated.

• Ski7 affects more lowly expressed genes than highly expressed genes.

• Show data suggesting that the genes are degraded in 3'-5' direction.

• Show that embryos lacking Ski7 are more resistant to reductive stress in vivo.

****Major comments:****

1.This manuscript is overall very nicely written but many of the conclusions are not convincing because they are not adequately supported because of a lack of appropriate controls. Most importantly, the authors do not appear to have controlled for potential off-target effects of the CRISPR method. Was more than one CRISPR line tested for the phenotype? Is fertilization rescued by overexpression of Ski7? This type of control is essential for the validity of the model and the resulting data and should be feasible without incurring substantial costs or time.
Thank you very much for bringing up this important point regarding the validity of our *ski7* mutant and the conclusions drawn from our phenotypic analysis of this mutant. We were aware of this weakness of our study and had already started generating a Ski7 rescue line to address whether the phenotypes observed were indeed due to lack of Ski7 (as opposed to some other background or off-target effect). At the time of submission, we did not have this line in the homozygous mutant background, yet have by now obtained homozygous mutant *ski7* fish that also contain the ubiquitously expressed Ski7-GFP transgene. Analysis of fertility of this line, compared to *ski7* homozygous mutants, revealed that fertility is fully rescued in the presence of the transgene. This allows us to make the strong conclusion that the fertility defect observed in our *ski7* mutants is indeed due to lack of Ski7. We have included this new data in Figure 1 (**Fig. 1D**) and updated the text and methods accordingly.

2.The authors never show that Ski7 protein is absent in the null fish. It does not appear on the list of downregulated genes in the TMT-MS in Sup. Fig. 8. Is there a Ski7 paralog, given the rather subtle phenotype?
Thanks for bringing up this point. In our TMT-MS experiments we have not been able to detect peptides that specifically originate from the Ski7-specific exon, neither in WT nor in mutants. However, we detected peptides originating from the shared region between Hbs1l/Ski7 with no differences between WT and *ski7*$^{-/-}$. We have highlighted Hbs1l in the **Sup. Data 5**, in which Hbs1l has a fold change of 1.1 and p-value of 0.376.
Hbs1l is a homologue of Ski7 that in yeast is encoded in two different loci. However, in vertebrates and plants, they are encoded as alternative splice isoforms. Although we do not know whether Hbs1l can compensate for possibly some functions of Ski7 in vertebrates, our data clearly shows that the decrease in fertility in the absence of Ski7 is Ski7-specific and cannot be compensated for by the presence of Hbs1l. Hbs1l lacks the interacting domain to the exosome, arguing against a possible compensation by Hbs1l. What we consider might be a potential compensatory pathway is the 5’-3’ RNA degradation machinery (Xrn1), as it has been shown to complement and genetically interact with the 3’-5’ degradation machinery components in yeast (Johnson AW & RD Kolonder, 1995).

3.The authors never show that the Ski7-null fish express normal levels of Hbs1l, which, based on its function in RNA decay, could also impact RNA levels.
Thank you for your comment. We added a panel in supplementary figure 1 **(Sup. Fig. 1C**)**,** which shows that the RNA levels of *hbs1l* in *WT* and mutants during the oocyte-to-embryo transition remain stable and don’t differ between *WT* and ski7 mutants. In addition, we have analyzed the change in Hbs1l

protein based on our TMT-MS data. The fold change between *ski7/WT* embryos based on TMT-MS is only 1.1 (p-value 0.376). We therefore have no indication for a difference in Hbs1l RNA or protein levels in *ski7* mutants. We have also added a comment to the text in this regard.

4.No experiments were done to determine why the eggs from Ski7-null females were less likely to be fertilized. Given that this is the major phenotype, it seems like an obvious question and the manuscript is weaker without such experiments, though these experiments could be complex and time-consuming.
We thank the reviewer for the suggestions and appreciate their understanding that, although relevant for the story, such experiments will be complex and time-consuming. The rescue of the fertility defect by transgenic Ski7 clearly shows that the phenotype we observe is real and due to lack of Ski7, yet given the time-constraints, we believe that further experiments go beyond the scope of this manuscript.

5.It is hard to interpret the meaning of the low percentages of shared genes in the different developmental time points of each main developmental stage (oocyte, egg, embryo). One possibility is that because many of the DEGs are expressed at a low level, the stochastic nature of the detection method influences the outcome more than for more highly expressed genes. Instead of comparing these data to that of all WT expressed genes, it should be compared to that of all WT expressed genes that are expressed at a similarly low level.
Thank you for raising this point. We agree with the reviewer that a better comparison is with expressed genes that have similar transcript levels as the DEGs. We repeated all analyses with a significantly deeper sequenced library size of all samples (see comments by reviewer 1), and compared DEGs with expression-matched and group-size (number of genes) matched unchanged genes. This re-analysis revealed that there is a significant overlap between DEGs within periods (oogenesis, eggs, embryos) **(Fig. 3E).** On the other hand, the lack of overlap of DEGs between different periods persists **(Fig. 3E, Sup. Fig. 6-8).** We have updated our text and figures accordingly.

6.The authors show data suggesting that Ski7 degrades RNAs in a 3'-5' direction. Although this finding would not be surprising given what is known about Ski7 function in yeast, it is very difficult to see this in the data presented in Figure 4. Similar data presented in Sup. Fig. 3 is easier to follow. However, differences in the degradation of the 5'UTR, CDS, and 3'UTR should be validated against a set of unchanged genes that are expressed at a similar low level as the up-regulated genes. At least some of these genes also should be validated with real time PCR given the low expression levels.
Thank you for this comment. We modified figure 4 and included the previous supplementary figure as a panel in the main figure (**Fig. 4C**). Additionally, in a separate analysis, we used the same number of expression-matched unchanged genes (in regard to either up-regulated genes or down-regulated genes) and calculated the ratio of density per gene body region. We observed very similar results as with all expressed genes (for your information, the results of this additional analysis is shown below).

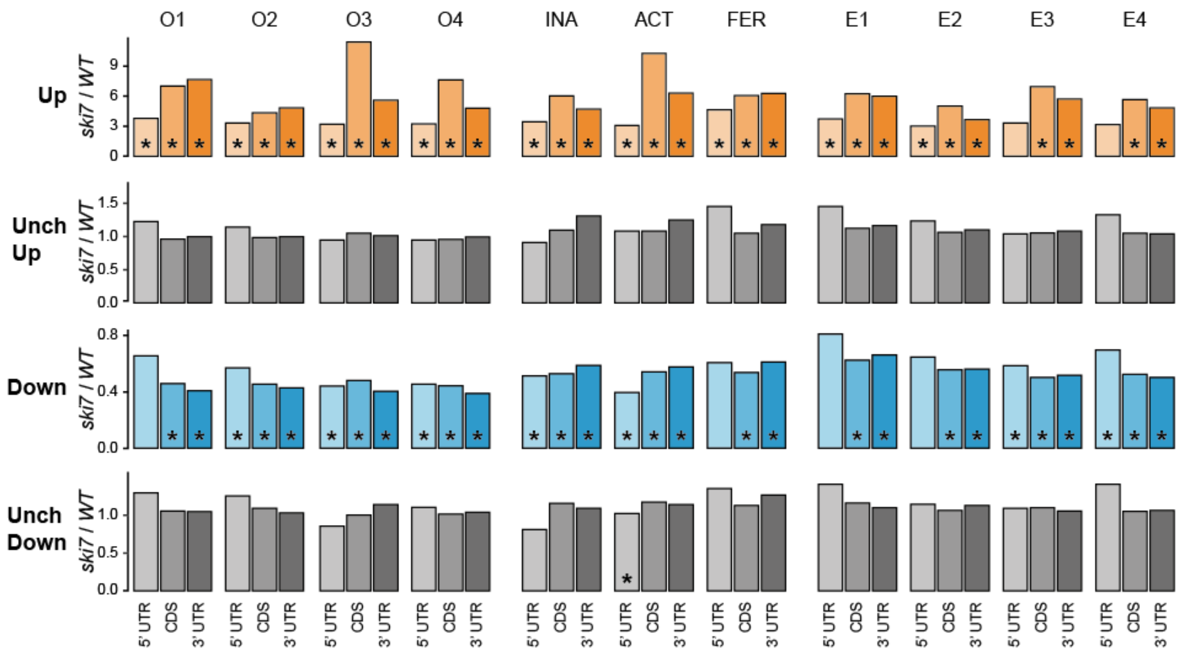

Additionally, we performed qPCR at time point E4 (sphere stage) for 3 up-regulated and 3 down-regulated selected genes to confirm our RNA-seq results. We observed similar trends (up- and down-regulated) when qPCR was performed. The graph below represents data from three different replicates.

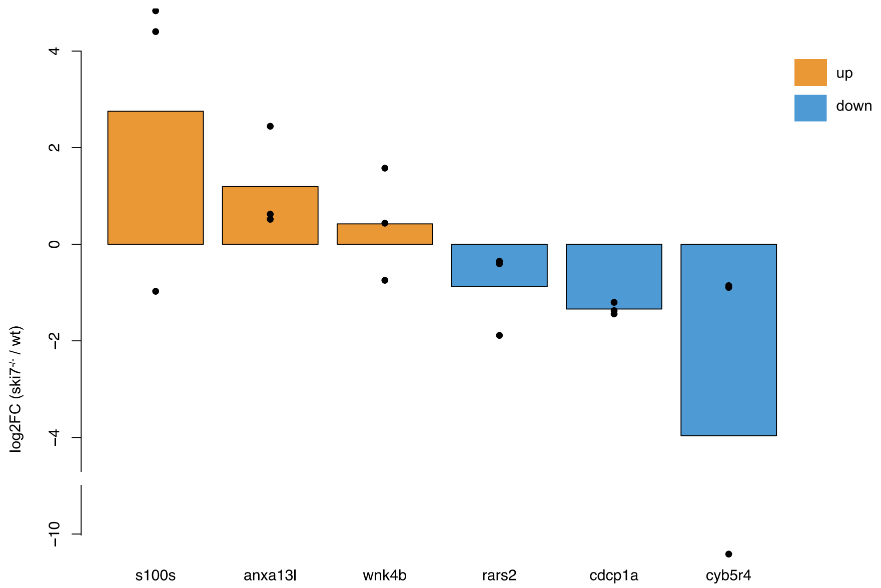

7.The RNAseq data for the WT inactive and WT fertilized eggs were from only 2 replicates for technical reasons. Unfortunately, this is a key time period for the function of Ski7, and some of the main conclusions of the manuscript are based on these RNAseq data. How do the authors draw conclusions regarding up- and down-regulated genes when there is an N=2 in one of the groups? What was the FDR cutoff for the genes called as up- or down-regulated?

We appreciate and fully agree with your comment regarding the statistical power of using only 2 replicates. We have now re-sequenced the two previously missing replicates (*WT* inactive, and *WT* fertilized) and included them in the analyses. In addition, we switched to EdgeR as a more conservative approach using an FDR of 0.05 to confidentially identify DEGs. With this new data, we repeated the analyses, and updated the text, figures, and methods accordingly.

Minor Comments:

1.Need to detail numbers of fish that generated oocytes/eggs/embryos for each experiment.
We added the number of embryos used for each experiment where appropriate. Thank you for pointing this out.

2.The description of the Statistical Analyses is inadequate. How were the specific tests chosen?
We have added a section in the methods describing the statistical tests used for every experiment or analyses performed. We apologize that this was not clear from the beginning.

3.Figure 1D - It is not clear which comparisons are used for the p-values above the 3 groups that have them in the graph.
We apologize for not being clear in the figure. We modified the figure 1D by adding the corresponding lines to indicate the type of comparisons performed. We also added a sentence in the figure legend of Sup. Fig. 3 to indicate that all the comparisons were performed against the cross of *wt* male with *wt* female.

4.Page 21 - "missed clavages"?
Thank you for checking this very carefully. We apologize for the spelling mistake and have corrected it to 'cleavages'.

5.The Supplemental Data tables are not described sufficiently to understand what was done. The Tables are not labeled with a title in the file name (though perhaps this is a function of the submission web site) or at the top of the table. There is no indication of what the colors mean. Why are there "imputed" normalized abundances used in Sup. Data. 3? Where is the description of how these data were analyzed?
We apologise for the unclear description of the data analysis. The imputation method was not specified in the original submission. We have added it in the revised version of the manuscript. We have also renamed the tables to indicate the type of the data sets as well as a header and a legend on every table. Moreover, we have included brief descriptions of the Supplemental Data in the revised version of the manuscript.

6.A Sup. Data set that showed lists of the differentially expressed genes and associated p-values and FDR values that are graphed in Figure 3D would be useful, despite the existence of the deposited data set.
Thank you for your suggestion. We included additional supplementary material including the list of differentially expressed genes at every time point (Supplemental Data 4).

7.Figure 3E is much more complex than necessary. The take home message is just the number of shared DEGs or expressed genes in each general stage of development. The authors never even refer to any of the additional numbers in the complicated Venn diagrams. Also, there is an error in the Egg Downregulated Venn diagram or %shared calculation, or both.
Thank you for your comment. We agree that the figure showed more information than what we actually mentioned in the text. We have modified the main figure and moved some information to the supplementary figures. Given our re-sequencing of all samples to increase the library depth to >10 Mio per sample, the numbers have been updated accordingly.

Reviewer #2 (Significance (Required)):

There is minimal information available regarding the function of Ski7 in vertebrates. There is a single

paper based on experiments in human cell lines finding that Ski7 (also known as HBS1LV3) interacts at the protein level with both the cytoplasmic exosome complex and the Ski complex, and functions in the same mRNA degradation pathway as observed in yeast (Kalisiak Nuc Acids Res 2017). Importantly, no specific cellular processes were affected, leading the authors to conclude that Ski7 has a general role in mRNA decay in humans.

Here the authors demonstrated a function for Ski7 in zebrafish fertilization in vivo, though they do not determine a mechanistic explanation for this finding. They also found a specific role for Ski7 in embryos - targeting for degradation mRNAs that regulate redox responses. This finding extends what was known in yeast and human to identify specific Ski7 targets, but does not develop this finding any further, e.g., by identifying specific motifs for target prediction.

Audience: These findings would be of interest to researchers who study early embryo development and how the environment impacts developmental success

Expertise: mammalian fertilization, egg activation, embryonic genome activation
Insufficient expertise: complex statistical analyses

REFEREES CROSS COMMENTING

I generally concur with the Major Comments provided by the other Reviewer, and appreciate the detail provided in the first point about the RNAseq analysis. I too had concerns about the RNAseq but did not express them so nicely.

For point #5 regarding the reductive stress effect, I'm not sure of the appropriate statistical analysis to be done given that the later time points all depend on the earlier time points. The authors don't indicate what test was done or why. It is not clear whether the embryos that appeared abnormal all remained abnormal or whether they regained a normal appearance. I, too, would appreciate more detail regarding how this experiment was performed and analyzed. Provision of untreated control and Ski7-null fish would strengthen the experiment, as mentioned by the other reviewer.
Thank you for your comments and apologies for the confusion. We added the untreated controls and performed Kruskal-Wallis with Dunn's multiple comparison per time point using *wt* untreated embryos as the reference. We consider that comparing survival of embryos per time point is appropriate as we quantify the defects in all conditions at the same time point.
