# Supplementary material for: Zebrafish Ski7 tunes RNA levels during the oocyte-to-embryo transition
Source: PLoS Genet. 2021 Feb 18;17(2):e1009390. doi: 10.1371/journal.pgen.1009390 (PMC7924785; doi:10.1371/journal.pgen.1009390)
Supplement: Attachment — Submitted filename: Rebuttal_CabreraQuio.docx [file pgen.1009390.s032.docx]

Thank you very much for submitting your Research Article entitled 'Zebrafish Ski7 tunes RNA levels during the oocyte-to-embryo transition' to PLOS Genetics. Since this was a manuscript transmitted from Review Commons, your manuscript was fully evaluated at the editorial level for its appropriateness at PLOS Genetics. The editors appreciated the strength of the transcriptomic analysis to examine the function of Ski7 in the oocyte to embryo transition in the zebrafish. The defect reported in fertility, however, requires further analysis prior to resubmission and review, as noted below. Additional more minor points below should also be addressed.

We request that analysis of fertilization itself be examined in the ski7 mutant, which is currently lacking. For example, does the sperm/male pronucleus enter the egg?  If it does enter, does pronuclear congression occur? If not, is the micropyle present or Bouncer affected?

We agree that it would be extremely interesting to determine whether zebrafish oocytes that lack Ski7 have compromised oocyte growth or maturation, or whether they are indeed fertilised, but zygotes fail to undergo further cell divisions. Based on the literature reported for other genes affecting remodelling of the transcriptome (*Cnot6l, MSY2, Ski complex* [Medevev, *et al.*, 2011; Sha, *et al.*, 2018; Horvat, *et al.*, 2018; Blatt, *et al.*, 2020]), we think that the subfertility phenotype observed in the ski7 mutants might be mainly due to defects during oocyte maturation.

We had noticed while experimenting with ski7 mutant fish a consistent trend that egg clutches laid by ski7 mutant females had a larger fraction of ‘bad quality’ eggs compared to egg clutches of wild-type siblings. We had not quantified this defect before yet have now performed additional crosses to quantify this phenotype. Since we think this is an important point to make, we decided to include this analysis in Sup. Fig. 3A. We have also changed the text accordingly to reflect that ski7 mutants present in fact two defects: 1) reduced quality of eggs; and 2) subfertility. The subfertility defect still holds true as an independent phenotype since for the analysis of fertility only normal-looking 1-cell stage embryos were kept and scored at 3-4 hpf for having developed into ~1000-cell stage embryos (= fertilized eggs).

Nevertheless, we agree with you that it would indeed be very interesting to identify the basis of the compromised fertility in ski7 mutants, and we have tried to address this point in two different manners:

1) Measuring of fertilization rates via staining of DNA shortly after fertilization: We have tried to distinguish fertilized eggs (those with three DAPI stained spots; one for the polar body, one for the female nucleus and one for the male nucleus) from unfertilized eggs (2 DAPI stained spots with only the polar body and female nucleus present) as done previously for our Bouncer paper (Herberg *et al*., 2018). Unfortunately, and as was also the case in our published Bouncer paper (Fig. 2A; Herberg *et al*., 2018), this experiment underestimates the real fertilization rate. In a wild-type cross, we were able to observe three DAPI-stained spots in only ~50% of fertilized 1-cell stage embryos, despite the fact that the observed IVF fertilization rate from the remaining half of the embryos that were allowed to develop further was >90%. Since the fertility defect of *ski7* mutants is, in contrast to infertile *bouncer* mutant females, only mild (~70% fertilization rate), these technical limitations of the assay do not allow us to address with the confidence that would be needed whether the sperm can actually enter the egg in the absence of Ski7. We currently do not know why the assay does not accurately report fertilization rates even in a wild-type situation – we have tried to replaced DAPI with the NuclearMask stain, tried with and without permeabilization and scoring at different times after IVF – yet none of these measures have resulted in an accurate depiction of the real fertilization rates that would allow us to distinguish in a manner that is accurate enough for the subtle fertility defect of ski7 mutants between sperm having entered the egg but the embryo arresting vs the sperm not having entered the egg.
2) Detecting the male-contributed DNA by PCR: The same problem applied when we attempted to detect a male-contributed transgene in individual 1-cell stage embryos by PCR. We were only able to detect the male-contributed transgenic marker in 2/16 1-cell stage embryos despite a fertilization rate of 94%. We are facing a sensitivity problem here since we are trying to detect a single copy of DNA in a very complex protein/lipid-rich sample.

Given that the fertility defect is only one aspect of our paper and the *ski7* mutant phenotype, we hope that you agree that – for the reasons outlined above – the more in-depth analysis of this phenotype goes beyond the scope of our main story. One of the reviewers (via ReviewCommons) supported such a view in saying that addressing this point will require “experiments that could be complex and time consuming”.

Your question regarding the level of Bouncer expression in ski7 mutant eggs is interesting, since we fully agree that if Bouncer was reduced in ski7 mutant eggs, this would point towards a defect in sperm entry given the known role of Bouncer in allowing sperm to enter. We therefore checked the expression level of *bouncer* RNA in our RNA-seq time-course data of WT and ski7 mutants. However, bouncer RNA levels are slightly higher in the ski7 mutant eggs and embryos than in wild-type (see graph below), yet not sufficiently different to be classified as a differentially expressed gene at any time point with our stringent criteria. As shown in Herberg, *et al.,* 2018, having higher levels of Bouncer (transgenic line) would – if at all - positively affect fertilization in zebrafish. Thus, the slight increase in the levels of bouncer mRNA cannot account for the subfertility defect of ski7 mutant fish.

Thank you also for asking about the micropyle. In wild-type eggs, every egg has a funnel-like structure, which comprises a pre-formed opening in the chorion through with the sperm can access the oolemma. We had not assessed *ski7* mutant eggs for the presence of this structure, yet have checked this now, following your suggestion since an absence of the micropyle in a subset of *ski7* mutant eggs could indeed be a possible reason why *ski7* mutant eggs fail to get fertilized. To this end, *ski7* mutant females were squeezed to obtain inactive eggs, which were individually observed under high magnification under a dissection scope. We were able to observe the presence of a micropyle in 100% of eggs from 2 wild-type females (24/24 and 26/26 eggs), and in 100% of eggs from 2 ski7 mutant females (25/25 and 39/39 eggs). A third female for each genotype had bad-quality eggs and could therefore not be scored since we noticed that the identification of the micropyle was dependent on the quality of the egg also in a wild-type situation (independently of the genotype we were unable to identify the micropyle structure in eggs of females with bad quality eggs).

Fertility decreases in older fish, so it is relevant to note if WT and transgene females used were of the same age as the mutant females?  Were the same males used for the WT and mutant females and what were their ages?  Were WT and transgene females used in the fertilization assays siblings to the ski7 mutant females to ensure a similar genetic background in the mixed TLAB strain used.

Thank you for your comment regarding possible age-related differences in fertility. We were aware of this concern and have taken care throughout of our experiments to match the ages of the fish analyzed, and whenever possible, used direct siblings as controls. For quantification of the fertilization rates we used fish that were between 5 months to 1 year old. We observed that the subfertility phenotype was present regardless the age of the fish. In addition, for the quantification of fertility, we used WT and ski7 mutant sibling fish (coming from the same parents). When outcrosses were performed to distinguish between male or female defects, we crossed wild-type sibling male or female to a *ski7* sibling mutant female or male, and when more crosses were used, age-matched males from a different stock were used. Finally, for fertility assays of the rescued line (transgenic Ski7-GFP line), we had only kept the transgenic embryos and not grown up the non-transgenic siblings, and thus used age-matched mutant fish lacking the transgene for comparison.

Clarify in Fig 5C if each dot represents a different female. If not, how many females were used and were the controls siblings of the same age? At least 3 different females of each strain should be used.

We apologize that the figure 5C was not explained sufficiently well. Every dot represents a different experiment comprising a pool of 20 to 50 embryos per condition, collected from 2 to 4 wild-type or *ski7* mutant pairs. We have modified the Materials and methods section to explain this procedure. The untreated controls were derived from the same batch of embryos, as collected embryos were split in half and analyzed in parallel. The age of the parent fish was between 6 months and 1 year.

In Fig 1B and 2A, please provide more specific information on the x-axis for the stages examined.

Thank you for pointing this out. We have modified the figure legends to indicate the stages used during each period.

Add in Methods how O3 and O2 oocytes were identified.

Thank you for your comment regarding the staging of the eggs. We have modified the Materials and methods section to describe in more detail how oocytes were classified into the different stages.

In addition to the comments suggested by the editor, we have made the following modifications in the main text and figures:

- Sup. Fig. 3: We added panel A with quantification of good and bad eggs, and also updated the text accordingly throughout the manuscript to indicate that ski7 mutants have in fact two defects (defect during oogenesis in producing good quality eggs; and defect in fertility). The previous graph in Sup. Fig. 3 was moved to panel B (here we now indicate with lines that the p-values correspond to the comparisons of every cross against wild-type crosses.
- We added a small part in the discussion section to indicate that we currently do not know why ski7^-/-^ mutant fish have reduce fertility.
- We changed “explanations” to “explanation” on page 10 (discussion).
- Funding section has been updated.
- Fig. 1C: the lines to underline the P4 and Ski7-like motifs were too thick, and it was hard to see the text. We therefore reduced the thickness of the lines.
- Fig. 3B; Fig. 3C: we have added the corresponding legend (circle for wild-type and triangle for *ski7^-/-^*) directly in the figure, as it was previously indicating only in the figure legend.
- Fig 5A; Sup. Fig 12A: X-axis in the volcano plot was extended.
- Fig. 5C: we wrongly had a duplicated image of a 2-cell embryo in wild-type untreated condition. We had replaced the image with the appropriate image at 2 hpf.
