# Supplementary material for: Zebrafish Ski7 tunes RNA levels during the oocyte-to-embryo transition
Source: PLoS Genet. 2021 Feb 18;17(2):e1009390. doi: 10.1371/journal.pgen.1009390 (PMC7924785; doi:10.1371/journal.pgen.1009390)
Supplement: Attachment — Submitted filename: Rebuttal_CabreraQuio.docx [file pgen.1009390.s033.docx]

Vienna, January 23 2021

Dear Mary, dear Reviewers,

Thank you for the overall very positive evaluation and for providing additional helpful comments. Below, we address the editorial comments and the feedback from the reviewers.

We hope that our explanations and the revisions we made to our manuscript make our manuscript ready for publication in PLOS Genetics.

We are looking forward to your response.

With best wishes,

Andrea Pauli

**Comments to the editor:**

Thank you very much for submitting your Research Article entitled 'Zebrafish Ski7 tunes RNA levels during the oocyte-to-embryo transition' to PLOS Genetics.

The manuscript was fully evaluated at the editorial level and by independent peer reviewers. The reviewers are very positive about the revised manuscript but identified some minor concerns that we ask you to address in a revised manuscript.

Additionally, the editors note that since fertility was not able to be addressed by assaying for fertilization, the sub-fertility defect is presumptive and not known. Other mutants in zebrafish are known to be fertilized but do not progress in development beyond the 1-cell stage, which could also be the case here. Such mutants do not affect fertility per se but instead are maternal-effect mutants. Please clarify this point in the text and then it can be stated to be a presumptive subfertility defect.

Thank you for your comment and for pointing out this valid concern. We have modified the text accordingly to clarify this point, and describe the defect no longer as ‘fertility phenotype’ but rather as ‘eggs do not develop beyond the one-cell stage’ or ‘presumptive fertility’ phenotype, as you suggested.

We therefore ask you to modify the manuscript according to the review recommendations. Your revisions should address the specific points made by each reviewer.

**Comments to the Authors:**

**Please note here if the review is uploaded as an attachment.**

Reviewer #1: The authors have nicely addressed the concerns I raised in the previous review, and the manuscript is far stronger as a result. In particular, the addition of a transgenic rescue and the increased numbers and depth of the RNAseq analysis were very important clearly described.

Thank you very much for the positive feedback!

However, I have one remaining concern with the text. I think it is misleading to describe the ski7 mutants as having two defects, reduced egg quality and subfertility. The authors provide no evidence that the subfertility is a function of anything other than a defect in egg quality. Eggs that do not appear “normal-looking” are clearly abnormal, but eggs that DO appear normal under a dissecting microscope may not be completely normal. Given that many eggs have reduced morphological quality it is likely that the normal appearing eggs are also impaired. The only way to truly answer this question would be to perform a timed degradation of ski7 mRNA or protein that did not impact ski7 levels in oocytes. Clearly this experiment would be beyond the scope of the current manuscript, but the text should be modified to accommodate this concern.

Thank you for raising this concern. We agree with the reviewer that with the current mutant and experiments performed, we cannot distinguish between the possibilities that a fraction of ‘normal-looking’ eggs is in fact already abnormal due to earlier defects during oogenesis, or possibly really a ‘normal egg’ up to the point of egg laying (and then specifically defective in either fertilization or early cell cleavage). As you pointed out, only an inducible degradation of Ski7 in the mature egg would allow to distinguish between these two scenarios. We agree that this would be a very interesting experiment to perform, yet as you already stated yourself, also think that this will go beyond the scope of this manuscript. We would like to point out that our RNA-seq experiments from mature eggs were performed on ‘normal-looking’ eggs, highlighting that differences at the molecular level are indeed also present in these normal-looking eggs. We have added a note in the methods to indicate that RNA-Seq on mature eggs was performed on ‘good quality’ eggs only.

There are typos on lines 584 and 585.

Thank you for highlighting these typos to us. We have corrected them in the text.

Reviewer #2: This is a revised manuscript and the authors have addressed the previous critiques through additional experiments and edits to the text. The findings are not supported by the data and the manuscript is suitable for publication.

Thank you very much for this positive feedback.

Reviewer #3: This is a really interesting manuscript describing the exosome-dependent remodeling of the maternal transcriptome in the female gamete. Specifically, by studying the function the adaptor factor Ski7 in zebrafish. This paper is highly significant in research on early vertebrate development as it is the first molecular and functional characterization of Ski7 in an animal system, which poses it as a new regulator of the oocyte to embryo transition. This work also provides clear background and directions for future studies.

The authors have obviously worked hard on this project and present a massive amount of interesting data in this study. Since first review, the clarity of the manuscript has improved a lot. The authors have put a great effort in addressing all the previous comments. The topic of post-transcriptional regulation during the oocyte to embryo transition is also very understudied in vertebrates and I applaud this group for ‘stepping up’ and attempting to tackle this topic. However, I feel there are a few comments that need to be addressed before publication.

Thank you very much for your enthusiastic feedback and useful comments.

Major comments:

1. Based on data presented in figure S3 the authors conclude that ‘bad eggs’, in figure (3), display an opaque phenotype. Are these eggs lysed? Can the authors examine this lysis phenotype? If yes, it needs to be examined in the unactivated, activated and collected from natural mating eggs. Also, it would be nice to examine whether the lysis phenotype is observed after scoring in the no clear cell and small chorion ones. This might help to classify ‘bad eggs’ as those associated with a gradually triggered lysis instead.

Thank you for raising this concern regarding our classification of ‘good’ and ‘bad’ quality eggs. We apologize for not having been clear enough what we mean with ‘eggs of poor quality’. We scored eggs within 20-30 minutes after natural crosses as ‘poor quality’ if they were opaque (‘black eggs’) or did not elevate their chorions. Amongst the ‘morphologically normal-looking eggs’ (‘good quality eggs’), a higher fraction of eggs of the ski7 mutant genotype than of wildtype siblings did not develop beyond the one-cell stage. In addition, when performing our dissections of ovaries for RNA-seq, we observed that a higher fraction of ski7 mutant oocytes of the different oogenesis stages was also of poor quality, suggesting that at least some oocytes already ‘lyse’ during oogenesis. We have not included this point in the paper since we have not performed quantitative analysis on this point and can thus only make a qualitative statement. On the other hand, the ‘normal-looking’ eggs that failed to be fertilized did not show any morphological defects at the one-cell stage (the chorion elevated normally; the eggs were clear). We have modified the text to describe the two phenotypes more clearly (egg quality phenotype and no development beyond the one-cell stage) and avoid misunderstandings. Moreover, we have changed the wording from ‘bad eggs’ to ‘poor quality eggs’ as this term has been used in the past, and have expanded the methods section on the scoring of embryo development and egg quality.

2. One conclusion raised by the authors is that physiological levels of redox signaling might be regulated by Ski7 during the oocyte to embryo transition. Given that transcriptomic and proteomic data is available for mutants, it would be relevant to assess whether Ski7 loss-of-function impacts in the expression of apoptosis-related genes likely involved in poor egg quality acquisition and reduced fertility. Also, and from the perspective of oocyte maturation, it would be important to assess whether ovarian steroidogenesis disruption would lead to up-regulated apoptosis by analyzing the expression of somatic genes (i.e cyp19a1a) promoting their synthesis in the mutant stage III (O3) and IV (O4) oocytes. This, assuming that some but not all follicle cells have been removed from oocytes after sorting. This might help to propose a model where the redox activity of apoptosis inducing factors could be impacting on egg quality and fertility, and lead the authors to hypothesize how Ski7 functions during oocyte maturation.

We thank the reviewer for these interesting questions and suggestions about checking the expression and regulation of apoptosis and steroidogenesis during oogenesis in *ski7* mutants. Our differential gene expression analyses during oogenesis do not provide any direct evidence that differentially expressed genes (DEGs) in the *ski7^-/-^* mutant females would have poor quality because of mis-regulation of either of those processes. First, no GO term associated with apoptosis or steroidogenesis were enriched in DEGs in the mutant. In addition, and based on your suggestion, we checked all mis-regulated genes during oogenesis and mature eggs for the presence of genes involved in apoptosis. Of the 132 genes from the zebrafish transcriptome with associated GO terms ‘apoptosis’, we did not find a significant number represented in our DEGs (Pearson chi square with continuity correction > 0.1 compared to apoptosis related genes in expressed genes at every time point) that belong to this category:

| Stage | Up | Down |
| --- | --- | --- |
| O1 | 6 | 4 |
| O2 | 7 | 4 |
| O3 | 6 | 4 |
| O4 | 4 | 2 |
| UNA | 6 | 6 |
| ACT | 7 | 6 |
| FER | 9 | 7 |

The upregulated genes in mature eggs were consistent in the three stages (UNA, ACT, FER) and were: *casp812, rb1, tp53, ddit4, sgk1,* and *prkcda*. Downregulated genes found in the three mature egg stages were *babam2, rybpb, rpl11, ypel3,* and *aktip*. Although we found several genes affected, more experiments would be required to specifically attribute the poor egg quality in *ski7* mutants to apoptosis mediated by those genes; we think this goes beyond the current manuscript.

In addition, we also specifically checked for DEGs involved in steroidogenesis. We used the 10 genes highlighted by Yazawa T, *et al* (2019) to be involved in ovarian steroidogenesis (*sf1, nr5a2, hsd3b2, cyp11a1, star, nr0b1, fdx1, fdxr, cyp17a1,* and *cyp19a1a*). We observed that only *hsd3b2* was upregulated during stages O3 and O4. Although upregulation of *hsd3b2* has been linked to prostate cancer, we do not know whether it would have an important role during oogenesis in zebrafish. Further experiments would be needed to look into this possibly interesting link, which we think goes beyond the current scope of the manuscript.

Finally, based on the paper you suggested for cross-checking genes mis-regulated in poor quality (PQ) eggs (Yilmaz, *et al* 2017), we used the list of 892 genes that were differentially expressed in PQ eggs compared to good quality (GQ) eggs. From those, we compared genes uniquely expressed or increased in expression in PQ to our list of upregulated genes in *ski7^-/-^* mutant oocytes and mature eggs. In addition, we used the list of genes that were uniquely expressed or increased in expression in good quality (GQ) eggs and compared it to our list of downregulated genes in *ski7^-/-^* mutant oocytes and mature eggs.

|  | Up in *ski7-/-* | | | | Down in *ski7-/-* | | | |
| --- | --- | --- | --- | --- | --- | --- | --- | --- |
|  | Unique PQ | | Increased PQ | | Unique GQ | | Increased GQ | |
|  | DEG | Exp | DEG | Exp | DEG | Exp | DEG | Exp |
| O1 | 1 | 71 | 1 | 96 | 3 | 57 | 6 | 178 |
| O2 | 1 | 71 | 1 | 96 | 3 | 57 | 7 | 179 |
| O3 | 1 | 71 | 1 | 95 | 5 | 56 | 6 | 180 |
| O4 | 1 | 71 | 0 | 97 | 2 | 57 | 3 | 180 |
| UNA | 6 | 55 | 5 | 88 | 6 | 51 | 15 | 173 |
| ACT | 7 | 55 | 5 | 86 | 5 | 50 | 13 | 171 |
| FER | 8 | 54 | 5 | 86 | 6 | 50 | 23 | 170 |

As can be observed from the results, few genes associated with PQ or GQ by Yilmaz and colleagues were found as DEGs in *ski7^-/-^* oocytes and mature eggs (Pearson chi square with continuity correction > 0.09 compared to the background expressed (Exp) genes).

In addition, we took advantage of an additional data set published by Cheung et al 2019, who performed transcriptomics of good and poor-quality eggs and identified 66 differentially regulated genes in poor quality eggs. We compared up-regulated genes in *ski7^-/-^* to up-regulated genes in poor-quality eggs, whilst down-regulated genes in *ski7^-/-^* were compared to down-regulated genes in poor-quality eggs. This analysis revealed very little overlap (only 1 upregulated gene was detected in O2 and 1 in O3; plus, the *rpf2* gene was found to be upregulated in all stages of mature eggs). Interestingly, some downregulated genes in poor-quality eggs were upregulated in *ski7^-/-^* mutants. These genes include ribosomal proteins or proteins associated with translation, suggesting that a lack of balance of translation-related genes rather than the direction of the mis-regulation (up or down) might affect egg quality. This has also been suggested by Rauwerda *et al* 2016.

To summarize these comparisons to published data sets, we have included a sentence in the discussion:

*“Furthermore, DEGs in ski7^-/-^ mutant oocytes and eggs partially overlapped with genes shown to be mis-regulated in poor-quality zebrafish eggs [44,45], further supporting our conclusion that mis-regulation of the transcriptome in ski7^-/-^ mutants might contribute to the poor quality of the eggs.”*

However, we would like to point out that none of these comparative analyses outlined above allows us to draw conclusions about possible causal relationships regarding the phenotype observed in *ski7^-/-^* mutants. Although we agree that it would be very interesting to be able to link the poor-quality egg phenotype to the redox mis-regulation phenotype during embryogenesis, we think that addressing this point will go beyond the scope of this manuscript.

3. Where is the Ski7 factor expressed? Given that a transgenic line is available, it would be nice to examine the expression of the Ski7 protein in somatic cells and oocytes. This might be able to clarify which ovarian cell type is associated with the oocyte maturation defect in the ski7 mutant.

Thank you very much for your comment regarding Ski7’s specific expression. We agree that knowing where Ski7 is expressed and at which time will further help to identify a specific role of Ski7 during oogenesis. Although we have the transgenic line tagged with sfGFP, we used a ubiquitous promoter (*act2b*) that was able to rescue the phenotype of ski7 mutants, but does not allow us to address this point.

Minor comments:

1. The causes and molecular predictors of zebrafish egg quality have also been described by others (i.e Yilmaz et al., 2017 (PLoS ONE); Cheung et al., 2019 (BMC Genomics)). It would be great if the authors discuss these papers.

Thank you for your interesting suggestions. We incorporated these and other references regarding egg quality into our discussion (please see also our comment above regarding the analysis performed).

2. Line 100: P4 should be defined here.

Thank you very much for your comment. We have added a brief explanation what P4 stands for (patch4-like motif).

3. Please change inactive to unactivated egg(s), and active to activated egg(s).

Thank you for your suggestions. We have now changed active and inactive appropriately in the text, figure legends, and figures. Consequently, we have also modified INA to UNA, to keep the abbreviation consistent.
